# Using *Caenorhabditis elegans* to Model Therapeutic Interventions of Neurodegenerative Diseases Targeting Microbe-Host Interactions

**DOI:** 10.3389/fphar.2022.875349

**Published:** 2022-04-28

**Authors:** Chenyin Wang, Chaogu Zheng

**Affiliations:** School of Biological Sciences, The University of Hong Kong, Hong Kong SAR, China

**Keywords:** neurodegenerative diseases, *Caenorhabditis elegans*, gut microbiome, curli fibers biofilm, csgA gene, Parkinson’s disease, disease modeling, microbe-host interaction

## Abstract

Emerging evidence from both clinical studies and animal models indicates the importance of the interaction between the gut microbiome and the brain in the pathogenesis of neurodegenerative diseases (NDs). Although how microbes modulate neurodegeneration is still mostly unclear, recent studies have started to probe into the mechanisms for the communication between microbes and hosts in NDs. In this review, we highlight the advantages of using *Caenorhabditis elegans* (C. elegans) to disentangle the microbe-host interaction that regulates neurodegeneration. We summarize the microbial pro- and anti-neurodegenerative factors identified using the *C. elegans* ND models and the effects of many are confirmed in mouse models. Specifically, we focused on the role of bacterial amyloid proteins, such as curli, in promoting proteotoxicity and neurodegeneration by cross-seeding the aggregation of endogenous ND-related proteins, such as α-synuclein. Targeting bacterial amyloid production may serve as a novel therapeutic strategy for treating NDs, and several compounds, such as epigallocatechin-3-gallate (EGCG), were shown to suppress neurodegeneration at least partly by inhibiting curli production. Because bacterial amyloid fibrils contribute to biofilm formation, inhibition of amyloid production often leads to the disruption of biofilms. Interestingly, from a list of 59 compounds that showed neuroprotective effects in *C. elegans* and mouse ND models, we found that about half of them are known to inhibit bacterial growth or biofilm formation, suggesting a strong correlation between the neuroprotective and antibiofilm activities. Whether these potential therapeutics indeed protect neurons from proteotoxicity by inhibiting the cross-seeding between bacterial and human amyloid proteins awaits further investigations. Finally, we propose to screen the long list of antibiofilm agents, both FDA-approved drugs and novel compounds, for their neuroprotective effects and develop new pharmaceuticals that target the gut microbiome for the treatment of NDs. To this end, the *C. elegans* ND models can serve as a platform for fast, high-throughput, and low-cost drug screens that target the microbe-host interaction in NDs.

## Introduction

Neurodegenerative diseases (NDs), such as Alzheimer’s disease (AD), Parkinson’s disease (PD), Huntington’s disease (HD), and Amyotrophic Lateral Sclerosis (ALS), are characterized at the cellular level by the aggregation of misfolded proteins into *ß*-sheet-rich amyloid deposits in neurons, the failure of cellular proteostasis machinery to clear out the aggregates, and mitochondrial dysfunction and energy crisis that eventually lead to neuronal death (Martin, 2012). Different types of NDs involve distinct misfolded proteins, which can not only self-propagate in a prion-like fashion but also recruit other types of proteins and convert them to misfolded conformers in a process called cross-seeding (e.g., Aβ cross-seeds the aggregation of α-synuclein) (Lim, 2019). The latter may explain the co-occurrence of multiple NDs in the same patient. Although great efforts have been devoted to developing therapeutics that can remove existing protein aggregates or prevent the formation of new ones, almost no drugs showed success in clinical trials for the treatment of NDs. Such failure suggests that our understanding of ND pathogenesis is likely incomplete, and new ideas are needed for therapeutic interventions.

One of such new ideas in the past decade came from the realization that intestinal bacteria may be a crucial predisposing factor that contributes to the development of ND through the ‘‘microbiota-gut-brain axis’’ (Peterson, 2020). ND patients have altered microbial composition in the gut compared to healthy individuals (Li C. et al., 2019). Gastrointestinal dysfunction and intestinal inflammation are positively correlated with an elevated risk of PD (Fasano et al., 2015; Chen et al., 2019), and infection with *Helicobacter pylori* has been associated with increased severity of PD (Tan et al., 2015; Huang HK. et al., 2018). Moreover, both clinical studies and laboratory research indicate that microbes and their metabolic products can cross the blood-brain barrier to cause chronic inflammation in the brain, which is an important risk factor for neurodegeneration in several NDs, such as AD (Cattaneo et al., 2017) and PD (Wang H. et al., 2020). Despite the emerging link between the gut microbiota and NDs, the development of therapeutics that target the microbe-host interaction in the treatment of NDs is still in its infancy, largely due to the limited mechanistic understanding of the communication between the microbiota and the host and the lack of high-throughput, physiologically relevant model systems to screen drug candidates for their therapeutic effects. In this review, we describe the use of *Caenorhabditis elegans* ND models to identify microbial components that affect neurodegeneration and to test chemical compounds for their potential effects in inhibiting neurodegeneration. Specifically, by closely examining the literature, we generated a comprehensive list of compounds that showed both neuroprotective effects in *C. elegans* ND models (oftentimes confirmed in mouse models) and inhibitory effects on bacterial biofilm formation. Our summary highlights the possibility of targeting the secretion of extracellular fibrous polymers (e.g., curli) by gut bacteria as a novel therapeutic strategy for NDs.

### *C. elegans* ND Models

Although mouse ND models provided crucial insights into the neurodegenerative symptoms associated with neuro- and systemic inflammation caused by abnormal microbiota or pathogenic bacteria infection, the complexity of the mammalian nervous system and microbiome often makes it difficult to pinpoint the key microbial proteins or metabolites that directly impact the host neurons in the progression of neurodegeneration. Thus, the use of simpler organisms, such as *C. elegans*, became instrumental in disentangling the microbe-host interaction in the context of NDs.

First, *C. elegans* uses bacteria as their natural diet, and alteration of the bacterial genomes has been shown to affect the development and behavior of *C. elegans* (Watson et al., 2014). Moreover, the microbiome of *C. elegans* in its natural habitats has been characterized and many of these bacteria can be cultured in the laboratory (Berg et al., 2016). Second, the presence of microorganisms can be effectively controlled using a bleaching method that kills all microbes but keeps the eggs unharmed, thus allowing the cultivation of *C. elegans* under monoxenic conditions or with a defined mixture of microbes. Third, the nematode is transparent, so that the interaction between microbes and fluorescently labeled neurons can be visualized in live animals. Fourth, many signaling molecules and pathways are evolutionarily conserved between *C. elegans* and humans, indicating that the disease mechanisms found in *C. elegans* could be conserved in humans. Fifth, *C. elegans* has a short life cycle and is highly amenable to genetic manipulations. Several transgenic *C. elegans* ND models have been generated and provided important insights into the genetic factors that contribute to NDs. These models were also used as drug testing platforms to evaluate the therapeutic potential of various chemical compounds and natural products. Since *C. elegans* ND models have been extensively reviewed elsewhere (Alexander et al., 2014; Van Pelt and Truttmann, 2020), we will only briefly mention the most widely used transgenic ND models and their use in drug discovery and focus more on the microbial factors of the diseases.

A general strategy to model human NDs in *C. elegans* is to express the human proteins that form the protein aggregates in *C. elegans* muscles or neurons and to observe the degenerative phenotypes and aggregation of fluorescently labeled proteins. For AD, the first *C. elegans* model expressed the human Aβ peptide in the body wall muscle and found an age-dependent paralysis phenotype (Link, 1995). This model, however, has several disadvantages. First, a signal peptide is cleaved in the process of Aβ peptide generation, which leads to the production of Aβ_3-42_ instead of the Aβ_1-42_ found in human patients (McColl et al., 2012). Second, the age-dependent paralysis phenotype came under scrutiny as it is unclear whether the phenotype is a result of Aβ toxicity or intrinsic aging. To overcome these problems, other *C. elegans* AD models were constructed to express full-length human Aβ peptides under the control of a temperature-sensitive mRNA surveillance system that induces Aβ production after heat shock (Link et al., 2003; Hassan et al., 2009). These studies were able to observe an early-onset paralysis phenotype caused by Aβ toxicity in the muscle of young adult animals. To model the neuropathology of human AD more closely, later studies expressed Aβ in *C. elegans* neurons using pan-neuronal promoters and found that these AD animals have a shorter lifespan, impaired associative learning, and a significant decrease in serotonin-stimulated egg-laying (Wu et al., 2006).

The microtubule-associated protein Tau, which forms the neurofibrillary tangles in AD, was also expressed in *C. elegans* to model AD. The expression of the human Tau (V337M) mutants under a pan-neuronal promoter recapitulated some of the key features of AD in *C. elegans*, including uncoordinated movement, accumulation of insoluble tau, and age-dependent neuronal degeneration and loss. Similarly, Miyasaka et al. (2005) established the second tau model by expressing Tau mutants in the mechanosensory neurons of *C. elegans*; this model showed accumulation of hyperphosphorylated tau, morphological alteration of these touch neurons, and a progressive decrease in their sensory functions. More recently, Aβ and Tau co-expression models were also generated in *C. elegans* and showed increased deficits in associative learning, enhanced neuronal loss, and caused specific transcriptomic changes, compared to the single transgenic models (Wang et al., 2018).

Similarly, for PD models, human α-synuclein was expressed in *C. elegans* body wall muscles or neurons. Pan-neuronal expression of α-synuclein (A53T) mutants but not the wild-type protein caused defects in locomotion and the loss of dopaminergic neurons, which recapitulated the major aspects of PD symptoms in humans. Using the *C. elegans* PD model, the Caldwell group identified genetic factors that affect α-synuclein-mediated proteotoxicity via genome-wide RNAi screen and uncovered the involvement of the endocytic pathway in ameliorating α-synuclein toxicity (Hamamichi et al., 2008; Kuwahara et al., 2008).

For HD, which is caused by the polyglutamine (polyQ) expansion in the human huntingtin protein (Htt), Htt fused with polyQ repeats of different lengths were expressed in the ASH sensory neurons, which mediate avoidance behaviors to chemo- and mechanosensory stimuli. The expression of Htt-Q150 led to weak neurotoxicity in ASH neurons, but the loss of a glutamine/proline-rich protein PQE-1 significantly enhanced polyQ repeats-induced neurodegeneration (Faber et al., 2002). Using the same model, later studies found that the loss of several histone deacetylases or mutations in H3K9 methyltransferases and H3K9 methylation readers also enhanced polyQ toxicity (Bates et al., 2006; Zheng et al., 2013). Pharmacological screens with the HD model identified the neuroprotective role of mithramycin (MTR), trichostatin A (TSA), and lithium chloride (LiCl) (Voisine et al., 2007).

ALS is characterized by progressive death of motor neurons and is associated with mutations in genes encoding the Cu/Zn superoxide dismutase 1 (SOD1), RNA-binding proteins TDP-43, and fused in sarcoma (FUS). Expression of SOD1 (G85R) mutants fused with GFP in *C. elegans* neurons resulted in the formation of insoluble SOD1 aggregates in the perinuclear region of motor neurons and strong locomotor defects (Wang et al., 2009). Similarly, human TDP-43 (A315T) mutants were expressed in *C. elegans* neurons and the loss of GABAergic motor neurons and the change in locomotion speed were used as phenotyping criteria to test compounds for their neuroprotective effects against TDP-43-mediated toxicity (Boyd et al., 2014). Using the *C. elegans* ALS model, the Parker group screened more than 4000 FDA approved compounds and identified methylene blue, an aggregation inhibitor of the phenothiazine class, as a potent suppressor of mutant TDP-43 and FUS-induced neurotoxicity (Vaccaro et al., 2012b; Vaccaro et al., 2013; Therrien and Parker, 2014). Moreover, Kraemer and colleagues showed that inhibition of cell division cycle 7-related protein kinase (CDC7) by the small molecule inhibitor PHA767491 could reduce TDP-43 phosphorylation and prevent TDP-43-triggered neurodegeneration in *C. elegans* ALS models (Liachko et al., 2013).

### Pro-Neurodegenerative Factors in Bacteria

Pioneering works from mouse models pointed out a pro-neurodegenerative role of the intestinal bacteria in PD animals. For example, antibiotic treatment ameliorates the pathophysiology of PD mice, and microbial recolonization after the treatment restored the PD symptoms (Sampson et al., 2016). Colonization of α-synuclein-overexpressing mice with the gut microbiota from PD patients exacerbated the physical impairments compared to transplantation of microbiota from healthy donors (Sampson et al., 2016). Metagenomic analysis of the fecal samples of PD patients revealed not only changes in gut bacterial composition (e.g., increased Lactobacillaceae and Akkermansiaceae, decreased *Faecalibacterium* and *Roseburia* (Barichella et al., 2019; Nishiwaki et al., 2020)), but also a decrease of total intestinal bacterial count compared to healthy controls (Hasegawa et al., 2015). Fecal microbiota transplantation (FMT) from healthy donors was able to alleviate the tremor and some gastrointestinal dysfunctions (e.g., constipation) in PD patients (Huang et al., 2019). Similarly, for AD, cognitive deficits, protein aggregation of Aβ and hyper-phosphorylation of tau, and synaptic plasticity were significantly improved after FMT in mouse models (Sun et al., 2019). Rapid improvement of cognitive functions in senior AD patients after FMT was reported in two independent clinical cases (Hazan, 2020; Park et al., 2021). Despite the promise, the application of FMT has its limitations due to safety concerns and the limited availability of donor microbiota. Targeted treatment of the gut microbiota in PD patients is still more desirable than the gross replacement of the microbial flora. Identification of pro-neurodegenerative factors in bacteria is the key to the development of such targeted therapy.

*C. elegans* ND models provide a powerful tool to systematically discover bacterial components that contribute to ND pathogenesis. Recently, using several *C. elegans* PD models, we screened the entire genome of *E. coli* to identify pro-neurodegenerative genes by feeding the *E. coli* single-gene knockout strains in the Keio library (Baba et al., 2006) individually to PD worms and searched for genes whose deletion led to alleviation of α-synuclein-induced locomotion defect and dopaminergic neuron death (Wang C. et al., 2021). From the 3,985 non-essential *E. coli* genes, we identified 38 pro-neurodegenerative genes, which fall into several genetic pathways including curli formation, lipopolysaccharide (LPS) production, lysozyme inhibition, adenosylcobalamin synthesis, oxidative stress response, metabolism, and energy homeostasis. These results suggest that a diverse array of bacteria components could promote neurodegeneration in the host.

Among the bacterial pro-neurodegenerative factors, the curli amyloid fibril has a prominent function in promoting α-synuclein aggregation through cross-seeding. Curli fibril is formed by the polymerization of the major curli subunit CsgA with the help of the membrane-bound subunit CsgB. Both CsgA and α-synuclein are enriched in *ß*-sheet structures, and our immunofluorescent study found that bacteria-secreted CsgA could enter *C. elegans* neurons and human neuroblastoma cells to seed the aggregation of α-synuclein (Wang C. et al., 2021). Although curli proteins from different bacterial species were known to cross-seed (Zhou et al., 2012), and purified CsgA was found to accelerate α-synuclein fibrilization *in vitro* (Sampson et al., 2020), our study provided strong evidence for *in vivo* cross-seeding between CsgA and α-synuclein in neurons. This cross-seeding appears to be bidirectional, since α-synuclein also facilitated the retention of CsgA in neurons. Removing *csgA* or *csgB* from the *E. coli* genome significantly reduced α-synuclein aggregation, rescued mitochondrial dysfunction and energy failure, and prevented the loss of dopaminergic neurons. In addition to promoting α-synuclein neurotoxicity in PD, curli also promoted the toxicity of Aβ, SOD1, and Htt-polyQ in *C. elegans* models of AD, ALS, and HD, respectively, likely through similar cross-seeding mechanisms (Wang C. et al., 2021). Thus, bacterial curli may have detrimental effects on a range of NDs.

The idea that amyloid proteins produced by the gut bacteria may cross-seed endogenous proteins, such as Aβ and α-synuclein, to promote neurodegeneration has been hypothesized before (Friedland, 2015) and independently validated in multiple ND models in recent studies. In addition to the *C. elegans* models, oral exposure to curli-producing *E. coli* enhanced α-synuclein deposition in the brain of aged rats (Chen et al., 2016); and colonizing germ-free mice with curli-producing *E. coli* exacerbated α-synuclein-induced motor impairment compared to colonization with mutant *E. coli* that did not produce curli (Sampson et al., 2020). Thus, the pro-neurodegenerative role of bacterial curli has been validated in multiple organisms. Targeting curli production in the gut may be a novel therapeutic approach to prevent or slow down the progression of NDs.

Besides bacterial amyloid proteins, microbial metabolites or small molecules could also promote host neurodegeneration. For example, Ray et al. (2014a), showed that an unidentified bacterial metabolite produced by *Streptomyces venezuelae* caused age- and dose-dependent neurodegeneration in *C. elegans* PD models and human SH-SY5Y neurons. This neurotoxic metabolite increased the level of ROS and damaged mitochondria, disrupted proteostasis, and enhanced the toxicity of aggregation-prone proteins in multiple *C. elegans* ND models (Martinez et al., 2015). Mechanistically, the metabolite acts upstream of the ubiquitin-proteasome system (UPS) and PINK (a PD-associated kinase) to regulate mitochondrial maintenance and autophagy; the well-known antioxidant glutathione (GSH) attenuated the metabolite-enhanced α-synuclein toxicity and proteasomal dysfunction (Martinez et al., 2015).

### Anti-Neurodegenerative Effect of Microbes

In addition to the pro-neurodegenerative effects, studies have also found that certain bacteria and their metabolites could protect against protein aggregation and neurotoxicity. For example, the probiotic *Bacillus subtilis* inhibited α-synuclein aggregation and removed preformed aggregates in a *C. elegans* PD model (Goya et al., 2020). Interestingly, both dividing vegetative cells and environmentally resistant spores could inhibit α-synuclein aggregation but act through two distinct mechanisms: spores act *via* the PHA-4/Foxa dietary restriction pathways and vegetative cells *via* DAF-16/FOXO. Similarly, *Bacillus subtilis* also reduced Aβ-induced paralysis and cognitive defects and extended lifespan in a *C. elegans* AD model (Cogliati et al., 2020). The neuroprotective effect of *B. subtilis* may be mediated by beneficial gut-associated biofilm formation, the quorum-sensing peptide, and metabolites (e.g., nitric oxide). These results offer promises of using probiotics to prevent or delay neurodegeneration and suggest that altering microbial composition in the gut through nutraceutical interventions may have beneficial effects on NDs.

Some bacteria-derived compounds were shown to have anti-neurodegenerative effects. For example, mithramycin is produced by *Streptomyces plicatus* and is used as an antineoplastic drug to treat cancer by inhibiting RNA synthesis. Mithramycin is found to inhibit polyQ-mediated neuronal death in *C. elegans* HD models (Voisine et al., 2007) and to enhance motor performance and extend survival in a mouse HD model (Ferrante et al., 2004). Thus, bacteria-produced compounds, if able to cross the blood-brain barrier, may directly modulate neurodegeneration.

Microbes could also metabolize other nutrients or chemicals to produce neuroprotective effects. For example, Guo et al. (2020) found that water-soluble extracts of the herb *Peganum harmala L.* (wild rue) can be metabolized by *E. coli* OP50 (the laboratory diet for *C. elegans*) into oligosaccharides, which protected against polyQ-induced motility and fertility deficiency in *C. elegans* HD models.

Outside of the standard ND models, bacteria were also found to protect against neurotoxicity caused by leaky ion channels. Utilizing a neurotoxic allele of the mechanosensitive sodium channel to generate an ND model, Urrutia et al. (2020) found that certain bacteria species, including *E. coli* HT115, *Comamonas aquatica*, *Pseudomonas aeruginosa*, *Stenotrophomonas humi*, and *Bacillus megaterium* could protect neurons from leaky channel-induced degeneration. Interestingly, this neuroprotection is partially dependent on the GABA (γ-aminobutyric acid) produced by the bacteria. Since decreased GABA is associated with motor dysfunction in PD patients (Gong et al., 2018), microbe-derived GABA may also help alleviate motor defects in PD.

### Neuroprotective Compounds That Inhibit Bacterial Growth and Biofilm Formation

Since bacteria can produce amyloid-forming proteins (e.g., curli produced by intestinal *Enterobacteriaceae* (Bian et al., 2000) and SpaP produced by *Streptococcus mutans* in the oral cavity (Guo et al., 2017)), which may enter neurons to cross-seed protein aggregation, one possible treatment or preventive measure of NDs would be to inhibit the production of amyloid fibril by the bacteria. To identify potential drug candidates that target this pathway, we compiled a list of 59 neuroprotective compounds that reduced neurotoxicity in *C. elegans* and mouse ND models and highlight the 34 compounds that also inhibited microbial growth or biofilm formation (Table 1; description of their neuroprotective effects are in Supplementary Table S1). Several compounds were also able to induce biofilm dispersal. Since the amyloid fibers are the major constituent of the extracellular matrix in biofilms, compounds that inhibit biofilm formation likely also reduce amyloid productions. Although the neuroprotective and antibiofilm effects of these compounds were mostly identified in separate studies, we attempt to make connections between these two seemingly independent effects and propose that these chemical agents may suppress neurodegeneration at least partly by inhibiting the microbial secretion of amyloid fibrils. Below, we list some examples of these potential therapeutic compounds based on their known effects on microorganisms.

**TABLE 1:** Neuroprotective compounds identified in *C. elegans* neurodegenerative disease models and confirmed in mouse models showed effects on microorganisms.

Compounds	Worm models		Mouse model		Known effects on the microorganism
	Disease	Reference	Disease	Reference	
Ginkgo biloba extract*	AD	Wu et al. (2006)	AD	Tchantchou et al. (2007)	Inhibit biofilm formation (Wu et al., 2016).
Caffeine*	AD	Dostal et al. (2010)	AD	Arendash et al. (2006); Eskelinen and Kivipelto. (2010)	Inhibit bacteria growth at high dose; inhibit biofilm formation and cause biofilm dispersal (Chakraborty et al., 2020; Sandlie et al., 1980).
Clioquinol	AD	Matlack et al. (2014)	AD	Grossi et al. (2009)	Inhibit fungal biofilm formation (You et al., 2018; You et al., 2020).
Curcumin*	AD	Alavez et al. (2011); Miyasaka et al. (2016)	AD	Lim et al. (2001) Begum et al. (2008)	Inhibit biofilm formation (Kali et al., 2016) and induce biofilm dispersal (Ding et al., 2017).
Ferulic acid*	AD	Wang et al. (2020b)	AD	Wang et al. (2021b)	Inhibit bacteria growth and inhibit biofilm formation (Borges et al., 2012; Takahashi et al., 2013); induce biofilm dispersal (Dasagrandhi et al., 2018).
Fluoxetine*	AD	Keowkase et al. (2010b)	AD	Huang et al. (2018b)	Modulate bacterial gut colonization and inhibit biofilm formation (Fung et al., 2019; Pelling et al., 2019).
Galanthamine	AD	Xin et al. (2013)	AD	Bhattacharya et al. (2014)	N/A
Glycitein	AD	Gutierrez-Zepeda et al. (2005)		N/A	Among the antibacterial components of Doenjang extracts (Lalouckova et al., 2021)
JAY2-22-33	AD	Keowkase et a. (2010a)		N/A	N/A
JWB1-84-1	AD	Keowkase et al. (2010a)	AD	Sood et al. (2007)	N/A
Quercetin*	AD	Regitz et al. (2014)	PD	Ay et al. (2017)	Inhibit biofilm formation (Memariani et al., 2019).
Rifampicin*	AD	Lublin et al. (2011)	AD	Umeda et al. (2018)	Antibiotic; inhibit biofilm formation (Verma et al., 2021).
Tannic acid*	AD	Lublin et al. (2011)	AD	Takashi Mori (2012)	Inhibit bacterial growth and biofilm formation (Dong et al., 2018); induce biofilm dispersal (Siddiquia, 2019).
Tetracycline*	AD	Diomede et al. (2010)	AD	Balducci et al. (2018)	Antibiotic; inhibit biofilm formation (Stone et al., 2002).
Thioflavin T*	AD	Gamir-Morralla et al. (2019)	AD	Sarkar et al. (2015)	Inhibit biofilm formation (Bondia et al., 2021).
Acetylcorynoline	AD, PD	Fu et al. (2014)		N/A	N/A
Bacitracin*	AD, PD	Lehtonen et al. (2016); Lublin et al. (2011)	PD	Koutzoumis et al. (2020)	Antibiotic; inhibit biofilm formation (Zaidi et al., 2020).
EGCG*	AD, PD	Abbas and Wink. (2010); Wang et al. (2021a)	ALS, PD, AD	Dragicevic et al. (2011); Koh et al. (2006); Zhou et al. (2018)	Inhibit biofilm formation and induce biofilm dispersal (Serra et al., 2016)
Valproic acid	AD, PD	Evason et al. (2008); Kautu et al. (2013)	PD	Kidd and Schneider. (2011)	Inhibit fungal growth and fungal biofilm formation (Singh et al., 2021).
Acetaminophen*	PD	Chen et al. (2021); Locke et al. (2008); Lublin et al. (2011)		Zhao et al. (2017)	Inhibit biofilm formation (Abidi et al., 2019).
Losartan	PD	Chen et al. (2021)	PD	Chen et al. (2021)	N/A
Rifabutin*	PD	Chen et al. (2021)	PD	Chen et al. (2021)	Inhibit bacterial biofilm and infection (Doub et al., 2020).
Spermidine	AD, PD	Buttner et al. (2014); Yang et al. (2020)	FTLD	Wang et al. (2012)	Promote biofilm formation (Hobley et al., 2017; Thongbhubate et al., 2021).
Metformin*	AD, PD, HD	Ahmad and Ebert. (2017); Saewanee et al. (2021); Sanchis et al. (2019)	AD, HD, PD	Farr et al. (2019); Patil et al. (2014); Sanchis et al. (2019)	Inhibit bacterial biofilm and quorum sensing (Abbas et al., 2017).
Icariin and its derivative icariside II*	AD, HD	Cai et al. (2011)	AD	Li et al. (2019b)	Inhibit biofilm formation (Coenye et al., 2012).
PBT2	AD, HD	Cherny et al. (2012); McColl et al. (2012)	AD, HD	Cherny et al. (2012); Sedjahtera et al. (2018)	Inhibit polymyxin-resistance of Gram-negative pathogens (De Oliveira et al., 2020).
Apomorphine	PD	Mocko et al. (2010)	AD	Himeno et al. (2011)	N/A
Baicalin*	PD	Ma et al. (2021)	AD	Zhang et al. (2013)	Antimicrobial activity; inhibit biofilm formation (Luo et al., 2017).
Bromocriptine	PD	Mocko et al. (2010)	PD	Ogawa et al. (1994)	N/A
Betulin*	PD	Tsai et al. (2017)	AD	Cho et al. (2016)	Inhibit biofilm formation (Viszwapriya et al., 2016).
Indoline and its derivative GW5074	PD	Liu et al. (2011)	HD	Chin et al. (2004)	Inhibit gram-positive bacteria growth (Clement Opoku-Temeng, 2017).
Ginsenoside*	PD	Chalorak et al. (2021)	AD	Zhang et al. (2021)	Antibiofilm activity; induce biofilm dispersion (Cao et al., 2019).
Lisuride	PD	Braungart et al. (2004)	PD	Laloux et al. (2008)	N/A
LRRK2-IN1	PD	Yao et al. (2013)	PD	Chen et al. (2018)	N/A
P7C3	PD	De Jesus-Cortes et al. (2012)	PD	Gu et al. (2018)	N/A
Rottlerin*	PD	Braungart et al. (2004)	PD	Zhang et al. (2007)	Inhibit bacterial quorum sensing and biofilm formation (Suresh et al., 2021).
Sorafenib and its derivative*	PD	Liu et al. (2011)	PD	Zhang et al. (2017)	Inhibit biofilm formation (Cui et al., 2019).
Tauroursodeoxycholic acid	PD	Ved et al. (2005)	PD	Cuevas et al. (2020)	N/A
TTT-3002	PD	Yao et al. (2013)		N/A	N/A
Celecoxib*	HD	Ching et al. (2011)	PD	Kaizaki et al. (2013)	Inhibit biofilm formation (Tzeng et al., 2020).
Lithium	HD	Voisine et al. (2007)	HD	Chiu et al. (2011)	Absorbed by biofilm polymer (Kurniawan, 2013).
Mithramycin	HD	Voisine et al. (2007)	HD	Ferrante et al. (2004)	Produced by bacteria (Pham et al., 2019).
ML346*	HD	Calamini et al. (2010)		N/A	Inhibit biofilm formation (Guan et al., 2022).
Oligomycin*	HD	Varma et al. (2007)		N/A	Antibiotic; clear established biofilm (Yamada et al., 2020).
Rotenone	HD	Varma et al. (2007)		Inden et al. (2011)	N/A
Salidroside*	HD	Xiao et al. (2014)	PD	Zhang et al. (2016)	Inhibit biofilm formation (Coenye et al., 2012).
Trichostatin A and other HDAC inhibitors	HD, PD	Bates et al. (2006); Voisine et al. (2007)	PD	Suo et al. (2015)	Inhibit fungal biofilm formation (Cécile Garnaud et al., 2016).
Azaperone or isoniazid	FTDP	McCormick et al. (2013)	FTDP	Crowe et al. (2020)	N/A
Perphenazine	FTDP	McCormick et al. (2013)		N/A	N/A
Trazodone	FTDP	McCormick et al. (2013)	FTDP	Halliday et al. ( 2017)	N/A
Zotepine	FTDP	McCormick et al. (2013)		N/A	Inhibit fungal biofilm formation (Siles et al., 2013).
Guanabenz	ALS	Vaccaro et al. (2013)	ALS	Vieira et al. (2015)	N/A
Propyl gallate*	ALS	Tauffenberger et al. (2013)	AD	Chan et al. (2016)	Inhibit biofilm formation (Kosuru et al., 2021).
Salubrinal	ALS	Vaccaro et al. (2013)	ALS	Saxena et al. (2009)	N/A
Trolox	ALS	Tauffenberger et al. (2013)	ALS	Rojas et al. (2015)	N/A
α-methyl-α-phenylsuccinimide	ALS	Wong et al. (2018)		N/A	N/A
Methylene blue*	ALS, FTDP	Fatouros et al. (2012); Vaccaro et al. (2012a); Vaccaro et al. (2012b)	FTDP	Hosokawa et al. (2012)	Visualize biofilm; inhibit biofilm formation; induce biofilm dispersal (Shaw et al., 2020; Wu et al., 2009).
PHA767491	ALS	Liachko et al. (2013)	ALS	Chung et al. (2020)	N/A
LDN-0130436	ALS	Boyd et al. (2014)		N/A	N/A

Asterisks (*) mark the compounds that could inhibit the bacterial biofilm formation. “N/A” means the effect of the compounds is not assessed.

#### Antibiotics

Several of the neuroprotective compounds are well-known antibiotics, including tetracycline, rifampicin, oligomycin, and bacitracin. As an example, tetracycline, the first glycylcycline antibiotic, inhibits protein synthesis by blocking the binding of aminoacyl tRNA to bacterial ribosomes and has been extensively used to treat infections of various microorganisms, including Gram-positive and Gram-negative bacteria, intracellular bacteria Chlamydiae, protozoan parasites, etc. (Chopra and Roberts, 2001). Interestingly, tetracycline was found to decrease Aβ aggregation and alleviate Aβ-induced paralysis phenotype and oxidative stress in *C. elegans* AD models (Diomede et al., 2010). Similarly, Balducci et al. (2018) found that long-term treatment of Doxy, a second-generation tetracycline, reduced the level of Aβ oligomers (18-mers) and significantly restored memory in a mouse AD model. Surprisingly, even an acute treatment of Doxy was sufficient to improve memory formation.

Although the exact mechanism for the neuroprotective function of antibiotics, such as tetracycline, is still unclear, it is reasonable to suspect that they suppress neurodegeneration at least partly by inhibiting bacterial growth in the gut microbiome, given the significance of the microbiota-gut-brain-axis in NDs. Therefore, the FDA-approved antibiotics can be potentially repurposed to treat NDs.

#### Inhibitors of Bacterial Biofilm Formation

Many of the neuroprotective compounds were found to inhibit bacterial biofilm formation, suggesting a potential link between bacterial biofilm and neurodegeneration. For example, the polyphenol epigallocatechin-3-gallate (EGCG), which is a natural compound found in green tea extract, has been well-known for its effects in reducing oxidative stress, inhibiting protein aggregation, and protecting against neurodegeneration in PD and AD (Singh et al., 2016). At the same time, EGCG also has broad-spectrum effects in inhibiting biofilm formation (Serra et al., 2016). It is, however, unclear whether these two functions are connected.

Our recent study disentangled these two functions by feeding PD *C. elegans* with bacteria pre-treated with EGCG (Wang C. et al., 2021). In this scenario, only the bacteria but not the neurons are treated by EGCG. We found that EGCG strongly inhibits curli production and biofilm formation in *E. coli* bacteria. Importantly, treating the bacteria alone with EGCG provides strong protection against α-synuclein-induced neurodegeneration, which is almost indistinguishable from the effects of treating both the bacteria and the PD animals. Therefore, the neuroprotective effects of EGCG may be largely due to its activities in inhibiting the curli expression and bacterial biofilm formation.

Another example came from the bioactive components of *Ginkgo biloba* extract. *G. biloba* has an extensive history of being used to treat dementia in traditional Chinese medicine. In *C. elegans* AD models, *G. biloba* extract and one of its components, ginkgolide A, was found to reduce Aβ oligomerization and deposition and inhibit Aβ-induced paralysis and chemotaxis defects (Wu et al., 2006). In a mouse AD model, the same extract also reduced Aβ toxicity, improved cognitive functions, and induced neurogenesis in the hippocampus (Tchantchou et al., 2007). These studies support the use of *G. biloba* extracts as neuroprotective agents. Interestingly, *G. biloba* extracts and ginkgolic acid could block biofilm formation in *E. coli* O157:H7, *Staphylococcus aureus*, *Salmonella* and *Listeria* and downregulate the expression of curli structural subunit *csgA* in *E. coli* K12 (Lee et al., 2014; Wu et al., 2016). Thus, just like EGCG, the natural products in *Ginkgo biloba* extract may also exert neuroprotective effects by inhibiting curli production in gut bacteria.

From our literature search, we found 27 compounds that inhibit both neurodegeneration and bacterial biofilm formation (compounds with asterisks in Table 1). The correlation between the two activities in these compounds deserves further investigation. We hypothesize that at least some of these compounds may suppress neurodegeneration by blocking the cross-seeding of the bacterial amyloid proteins with ND-associated aggregation-prone proteins. Nevertheless, we could not rule out the possibility that some compounds may exert neuroprotective effects through multiple mechanisms that also include the inhibition of ER stress and oxidative stress (see below).

#### Inhibitors of Fungal Biofilm Formation

Among the neuroprotective agents, a few have antifungal effects and could inhibit fungal biofilm formation. For example, clioquinol is an antifungal drug wildly used to treat skin infections such as infected eczema and athlete’s foot. Clioquinol inhibits *Candida albicans* biofilm formation in a dose-dependent manner by disrupting metal ion homeostasis (You et al., 2020). Unexpectedly, clioquinol was also found to promote the degradation of Aβ oligomers and rescue Aβ toxicity in a *C. elegans* AD model (Matlack et al., 2014). Similarly, clioquinol could reduce Aβ burden and reverse memory impairment in a mouse AD model (Grossi et al., 2009). These studies highlight the possibility, although not tested, that the neuroprotective effects of clioquinol may be connected to its activity in regulating metal ion metabolism and biofilm formation in the microbes.

### Bacterial Biofilm, ER Stress, and Oxidative Stress in NDs

At the cellular level, the mechanisms of neurological damage in NDs involve protein aggregation, mitochondrial dysfunction, oxidative stress, calcium homeostasis dysfunction, and neuroinflammation (Jellinger, 2010). The loss of cellular homeostasis often leads to the activation of the endoplasmic reticulum (ER) stress-triggered unfolded protein response (UPR) pathway and the impairment of the nuclear factor erythroid 2-related factor 2 (Nrf2)-antioxidant response element (ARE) pathway, which play vital roles in ND pathogenesis (Branca et al., 2017; Ren et al., 2021).

ER stress is induced by disturbances in the structure and function of the ER with the accumulation of misfolded proteins and alterations in the calcium homeostasis. For example, tau aggregates trigger abnormal interactions between ER proteins and the essential components of ER-associated degradation (ERAD) in AD brains, leading to ER stress (Meier et al., 2015). Conversely, overexpression of *xbp-1*, a major regulator of UPR, alleviated ER stress and protected dopaminergic neurons from α-synuclein-induced neurotoxicity (Ray et al., 2014b). Intriguingly, pathogenic bacterial biofilm was also found to induce host ER stress. For example, when forming host-associated biofilms, Group A Streptococcus (GAS), a human pathogen that causes a range of infections, could secrete streptolysins, which induce host ER-stress in both mammalian cells and an *in vivo* mouse model (Vajjala et al., 2019). Thus, inhibiting microbial biofilm formation may reduce ER stress and provide beneficial effects for neurons in ND patients. Indeed, many of the neuroprotective compounds (e.g., Salubrinal in Table 1) showed activities of both inhibiting biofilm formation and reducing ER stress.

Nrf2-ARE pathway, an indicator and regulator of oxidative stress, plays an important role in protecting neurons from degeneration in many NDs. Reduced Nrf2 levels were found in human AD and PD brains and in animal models of AD (Branca et al., 2017; Ramsey et al., 2007). Removing Nrf2 increased the levels of Aβ and phosphorylated tau and enhanced neurodegeneration in a mouse AD model (Branca et al., 2017; Rojo et al., 2018), whereas activating Nrf2 (by knocking down its negative regulator) led to the reduction in oxidative stress and neuroinflammation (Williamson et al., 2012). Several compounds in our list (e.g., metformin and caffeine in Table 1) were shown to pharmacologically activate Nrf2, induce the expression of antioxidant enzymes, and protect neurons against degeneration (Link et al., 2003; Dostal et al., 2010; Boettler et al., 2011; Cui et al., 2016; Saewanee et al., 2021). For example, in a *C. elegans* model of AD, caffeine induced the nuclear translocation of SKN-1 (the *C. elegans* homolog of Nrf2) and delayed Aβ-mediated paralysis (Dostal et al., 2010). Given that these compounds also inhibit the formation of bacterial biofilms, it is unclear whether they suppress neurodegeneration by inhibiting cross-seeding or inducing antioxidative response or both. Reduced protein aggregation by the inhibition of cross-seeding may also facilitate the activation of the antioxidant Nrf2-ARE pathway.

Oxidative stress often exacerbates ER stress in NDs. During oxidative stress, the accumulation of reactive oxygen species (ROS) disrupts the redox-dependent protein folding process and thus increases the production of misfolded proteins, which further enhance ER stress and proteotoxicity in neurons. Alleviating both ER stress and oxidative stress provide synergistic benefit for the treatment of NDs. For example, curcumin, a polyphenol compound from the curry spice turmeric, possesses potent antioxidant and anti-UPR activities and could modulate multiple targets implicated in the pathogenesis of NDs (Lim et al., 2001; Begum et al., 2008). In fact, curcumin was shown to alleviate Aβ and tau-induced neurotoxicity and protein aggregation in *C. elegans* AD models (Alavez et al., 2011; Miyasaka et al., 2016).

Interestingly, curcumin shows anti-bacterial activity against a variety of infections when administrated together with antibiotics (Kali et al., 2016). Curcumin can inhibit biofilm formation, perturb bacterial membranes, disturb bacterial cell division, and alter gene expression patterns (Vaughn et al., 2017). Thus, although it is unclear whether the neuroprotective effect of curcumin relates to its bactericidal activity, this example raises the possibility of targeting bacterial biofilm to simultaneously reduce both ER stress and oxidative stress in NDs.

### Discussion and Future Perspectives

The gut microbiome holds the promise of becoming the therapeutic target of NDs, which currently have no effective treatments. Understanding the molecular mechanisms by which intestinal bacteria modulate neurodegeneration is, however, challenging, given the complexity of the microbial composition in the gut and the difficulties of studying the effects of a single bacterial component in isolation in a well-controlled system. Therefore, the use of simple model organisms like *C. elegans* can provide unparalleled advantages in studying the communication between microbes and neurons in the context of NDs. As we have shown above, using a variety of *C. elegans* ND models, both pro- and anti-neurodegenerative factors can be identified from the bacteria, paving the way for a mechanistic understanding of how bacterial proteins and metabolites affect host neurodegeneration.

Nevertheless, the *C. elegans* ND models also have certain limitations compared to rodent models. For example, *C. elegans* lacks the complex immune system found in mammals. Although certain molecular pathways in innate immunity are conserved between *C. elegans* and humans (Ermolaeva and Schumacher, 2014), *C. elegans* has no specialized immune cells, no adaptive immunity, and no typical inflammatory response. Thus, it will be difficult to use *C. elegans* models to investigate the effects of the microbes in triggering neuroinflammation. Interestingly, *C. elegans* does have glia cells comparable to mammalian microglia, but their roles in neurodegeneration have not been studied. The absence of a complex immune system in *C. elegans* ND models, however, simplifies the studies of microbe-neuron interaction and allows direct molecular interaction to be revealed.

In this review, we paid specific attention to bacterial amyloid proteins and biofilm formation as an important pro-neurodegenerative mechanism in microbes, given the cross-seeding between bacterial amyloid proteins (e.g., curli) and human endogenous aggregation-prone proteins (e.g., α-synuclein), both of which are enriched in *ß*-sheet structures. Guided by this cross-seeding mechanism, we searched the literature to identify compounds that showed both neuroprotective effects in ND models and antibiofilm effects on microorganisms and raised the hypothesis that some of these therapeutic compounds may suppress neurodegeneration at least partly through inhibiting bacterial amyloid production (which leads to antibiofilm activities) and thus preventing cross-seeding.

Although direct evidence demonstrating the causal relationship between the antibiofilm and neuroprotective activities are still missing for most compounds except for a few (e.g., EGCG (Wang C. et al., 2021), 30 (51%) out of the 59 neuroprotective compounds we found have known effects of inhibiting bacterial growth or biofilm formation, suggesting that the correlation of these two activities is quite strong. The percentage may be even higher, given that the effects on microorganisms are not tested for many of these compounds. Although the list we compiled (Table 1) is in no way a complete list, we hope it could inspire fellow researchers to consider the alteration of gut microbiota as a possible pharmacological mechanism of neuroprotective agents or to develop drugs that specifically target the intestinal microbes for treating NDs.

In fact, previous works have identified a wide range of anti-biofilm agents including FDA-approved drugs (Gilbert-Girard et al., 2020) and novel compounds (Junker and Clardy, 2007; Paytubi et al., 2017). It will be of great interest to test their neuroprotective effects with the attempts of repurposing them for the treatment of human NDs in future research. Again, the *C. elegans* ND models could be instrumental for screening these compounds for potential anti-neurodegenerative activities, given the convenience of setting up fast and high-throughput drug screens using *C. elegans*. Moving forward, with a deeper understanding of the mechanisms underlying the microbiota-gut-brain interactions in NDs and more therapeutic candidates targeting the gut microbiome for ND treatment, we expect a potential paradigm shift in the research of ND pathogenesis and drug development.

## References

[B1] Abbas H. A. Elsherbini A. M. Shaldam M. A. (2017). Repurposing Metformin as a Quorum Sensing Inhibitor in *Pseudomonas aeruginosa* . Afr. Health Sci. 17, 808–819. 10.4314/ahs.v17i3.24 29085409 PMC5656202

[B2] Abbas S. Wink M. (2010). Epigallocatechin Gallate Inhibits Beta Amyloid Oligomerization in *Caenorhabditis elegans* and Affects the Daf-2/insulin-like Signaling Pathway. Phytomedicine 17, 902–909. 10.1016/j.phymed.2010.03.008 20382008

[B3] Abidi S. H. Ahmed K. Kazmi S. U. (2019). The Antibiofilm Activity of Acetylsalicylic Acid, Mefenamic Acid, Acetaminophen against Biofilms Formed by *P. aeruginosa* and S. Epidermidis. J. Pak Med. Assoc. 69, 1493–1495. 10.5455/jpma.295488 31622303

[B4] Ahmad W. Ebert P. R. (2017). Metformin Attenuates Aβ Pathology Mediated through Levamisole Sensitive Nicotinic Acetylcholine Receptors in a *C. elegans* Model of Alzheimer's Disease. Mol. Neurobiol. 54, 5427–5439. 10.1007/s12035-016-0085-y 27596506

[B5] Alavez S. Vantipalli M. C. Zucker D. J. Klang I. M. Lithgow G. J. (2011). Amyloid-binding Compounds Maintain Protein Homeostasis during Ageing and Extend Lifespan. Nature 472, 226–229. 10.1038/nature09873 21451522 PMC3610427

[B6] Alexander A. G. Marfil V. Li C. (2014). Use of *Caenorhabditis elegans* as a Model to Study Alzheimer's Disease and Other Neurodegenerative Diseases. Front. Genet. 5, 279. 10.3389/fgene.2014.00279 25250042 PMC4155875

[B7] Arendash G. W. Schleif W. Rezai-Zadeh K. Jackson E. K. Zacharia L. C. Cracchiolo J. R. (2006). Caffeine Protects Alzheimer's Mice against Cognitive Impairment and Reduces Brain Beta-Amyloid Production. Neuroscience 142, 941–952. 10.1016/j.neuroscience.2006.07.021 16938404

[B8] Ay M. Luo J. Langley M. Jin H. Anantharam V. Kanthasamy A. (2017). Molecular Mechanisms Underlying Protective Effects of Quercetin against Mitochondrial Dysfunction and Progressive Dopaminergic Neurodegeneration in Cell Culture and MitoPark Transgenic Mouse Models of Parkinson's Disease. J. Neurochem. 141, 766–782. 10.1111/jnc.14033 28376279 PMC5643047

[B9] Baba T. Ara T. Hasegawa M. Takai Y. Okumura Y. Baba M. (2006). Construction of *Escherichia coli* K-12 In-Frame, Single-Gene Knockout Mutants: the Keio Collection. Mol. Syst. Biol. 2, 2006–0008. 10.1038/msb4100050 PMC1681482 16738554

[B10] Balducci C. Santamaria G. La Vitola P. Brandi E. Grandi F. Viscomi A. R. (2018). Doxycycline Counteracts Neuroinflammation Restoring Memory in Alzheimer's Disease Mouse Models. Neurobiol. Aging 70, 128–139. 10.1016/j.neurobiolaging.2018.06.002 30007162

[B11] Barichella M. Severgnini M. Cilia R. Cassani E. Bolliri C. Caronni S. (2019). Unraveling Gut Microbiota in Parkinson's Disease and Atypical Parkinsonism. Mov Disord. 34, 396–405. 10.1002/mds.27581 30576008

[B12] Bates E. A. Victor M. Jones A. K. Shi Y. Hart A. C. (2006). Differential Contributions of *Caenorhabditis elegans* Histone Deacetylases to Huntingtin Polyglutamine Toxicity. J. Neurosci. 26, 2830–2838. 10.1523/JNEUROSCI.3344-05.2006 16525063 PMC6675170

[B13] Begum A. N. Jones M. R. Lim G. P. Morihara T. Kim P. Heath D. D. (2008). Curcumin Structure-Function, Bioavailability, and Efficacy in Models of Neuroinflammation and Alzheimer's Disease. J. Pharmacol. Exp. Ther. 326, 196–208. 10.1124/jpet.108.137455 18417733 PMC2527621

[B14] Berg M. Stenuit B. Ho J. Wang A. Parke C. Knight M. (2016). Assembly of the *Caenorhabditis elegans* Gut Microbiota from Diverse Soil Microbial Environments. ISME J. 10, 1998–2009. 10.1038/ismej.2015.253 26800234 PMC5029150

[B15] Bhattacharya S. Haertel C. Maelicke A. Montag D. (2014). Galantamine Slows Down Plaque Formation and Behavioral Decline in the 5XFAD Mouse Model of Alzheimer's Disease. Plos One 9, e89454. 10.1371/journal.pone.0089454 24586789 PMC3931790

[B16] Bian Z. Brauner A. Li Y. Normark S. (2000). Expression of and Cytokine Activation by *Escherichia coli* Curli Fibers in Human Sepsis. J. Infect. Dis. 181, 602–612. 10.1086/315233 10669344

[B17] Boettler U. Sommerfeld K. Volz N. Pahlke G. Teller N. Somoza V. (2011). Coffee Constituents as Modulators of Nrf2 Nuclear Translocation and ARE (EpRE)-dependent Gene Expression. J. Nutr. Biochem. 22, 426–440. 10.1016/j.jnutbio.2010.03.011 20655719

[B18] Bondia P. Flors C. Torra J. (2021). Boosting the Inactivation of Bacterial Biofilms by Photodynamic Targeting of Matrix Structures with Thioflavin T. Chem. Commun. 57, 8648–8651. 10.1039/d1cc03155d 34369943

[B19] Borges A. Saavedra M. J. Simões M. (2012). The Activity of Ferulic and Gallic Acids in Biofilm Prevention and Control of Pathogenic Bacteria. Biofouling 28, 755–767. 10.1080/08927014.2012.706751 22823343

[B20] Boyd J. D. Lee P. Feiler M. S. Zauur N. Liu M. Concannon J. (2014). A High-Content Screen Identifies Novel Compounds that Inhibit Stress-Induced TDP-43 Cellular Aggregation and Associated Cytotoxicity. J. Biomol. Screen. 19, 44–56. 10.1177/1087057113501553 24019256 PMC3913261

[B21] Branca C. Ferreira E. Nguyen T. V. Doyle K. Caccamo A. Oddo S. (2017). Genetic Reduction of Nrf2 Exacerbates Cognitive Deficits in a Mouse Model of Alzheimer's Disease. Hum. Mol. Genet. 26, 4823–4835. 10.1093/hmg/ddx361 29036636 PMC5886066

[B22] Braungart E. Gerlach M. Riederer P. Baumeister R. Hoener M. C. (2004). *Caenorhabditis elegans* MPP+ Model of Parkinson's Disease for High-Throughput Drug Screenings. Neurodegener Dis. 1, 175–183. 10.1159/000080983 16908987

[B23] Büttner S. Broeskamp F. Sommer C. Markaki M. Habernig L. Alavian-Ghavanini A. (2014). Spermidine Protects against α-synuclein Neurotoxicity. Cell Cycle 13, 3903–3908. 10.4161/15384101.2014.973309 25483063 PMC4614020

[B24] Cai W. J. Huang J. H. Zhang S. Q. Wu B. Kapahi P. Zhang X. M. (2011). Icariin and its Derivative Icariside II Extend Healthspan via insulin/IGF-1 Pathway in *C. elegans* . Plos One 6, e28835. 10.1371/journal.pone.0028835 22216122 PMC3244416

[B25] Calamini B. Silva M. C. Madoux F. Hutt D. M. Khanna S. Chalfant M. A. (2010). “ML346: A Novel Modulator of Proteostasis for Protein Conformational Diseases,” in Probe Reports from the NIH Molecular Libraries Program. 23833797

[B26] Cao X. Ye Q. Fan M. Liu C. (2019). Antimicrobial Effects of the Ginsenoside Rh2 on Monospecies and Multispecies Cariogenic Biofilms. J. Appl. Microbiol. 126, 740–751. 10.1111/jam.14178 30556937

[B27] Cattaneo A. Cattane N. Galluzzi S. Provasi S. Lopizzo N. Festari C. (2017). Association of Brain Amyloidosis with Pro-inflammatory Gut Bacterial Taxa and Peripheral Inflammation Markers in Cognitively Impaired Elderly. Neurobiol. Aging 49, 60–68. 10.1016/j.neurobiolaging.2016.08.019 27776263

[B28] Chakraborty P. Dastidar D. G. Paul P. Dutta S. Basu D. Sharma S. R. (2020). Inhibition of Biofilm Formation of *Pseudomonas aeruginosa* by Caffeine: a Potential Approach for Sustainable Management of Biofilm. Arch. Microbiol. 202, 623–635. 10.1007/s00203-019-01775-0 31773197

[B29] Chalorak P. Sanguanphun T. Limboonreung T. Meemon K. (2021). Neurorescue Effects of Frondoside A and Ginsenoside Rg3 in *C. elegans* Model of Parkinson's Disease. Molecules 26. 10.3390/molecules26164843 PMC8402114 34443430

[B30] Chan S. Kantham S. Rao V. M. Palanivelu M. K. Pham H. L. Shaw P. N. (2016). Metal Chelation, Radical Scavenging and Inhibition of Aβ₄₂ Fibrillation by Food Constituents in Relation to Alzheimer's Disease. Food Chem. 199, 185–194. 10.1016/j.foodchem.2015.11.118 26775960

[B31] Chen J. Chen Y. Pu J. (2018). Leucine-Rich Repeat Kinase 2 in Parkinson's Disease: Updated from Pathogenesis to Potential Therapeutic Target. Eur. Neurol. 79, 256–265. 10.1159/000488938 29705795

[B32] Chen K. S. Menezes K. Rodgers J. B. O'Hara D. M. Tran N. Fujisawa K. (2021). Small Molecule Inhibitors of α-synuclein Oligomers Identified by Targeting Early Dopamine-Mediated Motor Impairment in *C. elegans* . Mol. Neurodegener 16, 77. 10.1186/s13024-021-00497-6 34772429 PMC8588601

[B33] Chen Q. Q. Haikal C. Li W. Li J. Y. (2019). Gut Inflammation in Association with Pathogenesis of Parkinson's Disease. Front. Mol. Neurosci. 12, 218. 10.3389/fnmol.2019.00218 31572126 PMC6753187

[B34] Chen S. G. Stribinskis V. Rane M. J. Demuth D. R. Gozal E. Roberts A. M. (2016). Exposure to the Functional Bacterial Amyloid Protein Curli Enhances Alpha-Synuclein Aggregation in Aged Fischer 344 Rats and *Caenorhabditis elegans* . Sci. Rep. 6, 34477. 10.1038/srep34477 27708338 PMC5052651

[B35] Cherny R. A. Ayton S. Finkelstein D. I. Bush A. I. McColl G. Massa S. M. (2012). PBT2 Reduces Toxicity in a *C. elegans* Model of polyQ Aggregation and Extends Lifespan, Reduces Striatal Atrophy and Improves Motor Performance in the R6/2 Mouse Model of Huntington's Disease. J. Huntingtons Dis. 1, 211–219. 10.3233/JHD-120029 25063332

[B36] Chin P. C. Liu L. Morrison B. E. Siddiq A. Ratan R. R. Bottiglieri T. (2004). The C-Raf Inhibitor GW5074 Provides Neuroprotection *In Vitro* and in an Animal Model of Neurodegeneration through a MEK-ERK and Akt-independent Mechanism. J. Neurochem. 90, 595–608. 10.1111/j.1471-4159.2004.02530.x 15255937

[B37] Ching T. T. Chiang W. C. Chen C. S. Hsu A. L. (2011). Celecoxib Extends *C. elegans* Lifespan via Inhibition of Insulin-like Signaling but Not Cyclooxygenase-2 Activity. Aging Cell 10, 506–519. 10.1111/j.1474-9726.2011.00688.x 21348927 PMC3094508

[B38] Chiu C. T. Liu G. Leeds P. Chuang D. M. (2011). Combined Treatment with the Mood Stabilizers Lithium and Valproate Produces Multiple Beneficial Effects in Transgenic Mouse Models of Huntington's Disease. Neuropsychopharmacology 36, 2406–2421. 10.1038/npp.2011.128 21796107 PMC3194069

[B39] Cho N. Kim H. W. Lee H. K. Jeon B. J. Sung S. H. (2016). Ameliorative Effect of Betulin from Betula Platyphylla Bark on Scopolamine-Induced Amnesic Mice. Biosci. Biotechnol. Biochem. 80, 166–171. 10.1080/09168451.2015.1072460 26287580

[B40] Chopra I. Roberts M. (2001). Tetracycline Antibiotics: Mode of Action, Applications, Molecular Biology, and Epidemiology of Bacterial Resistance. Microbiol. Mol. Biol. Rev. 65, 232–contents. 10.1128/MMBR.65.2.232-260.2001 11381101 PMC99026

[B41] Chung Y. H. Lin C. W. Huang H. Y. Chen S. L. Huang H. J. Sun Y. C. (2020). Targeting Inflammation, PHA-767491 Shows a Broad Spectrum in Protein Aggregation Diseases. J. Mol. Neurosci. 70, 1140–1152. 10.1007/s12031-020-01521-y 32170713

[B42] Coenye T. Brackman G. Rigole P. De Witte E. Honraet K. Rossel B. (2012). Eradication of Propionibacterium Acnes Biofilms by Plant Extracts and Putative Identification of Icariin, Resveratrol and Salidroside as Active Compounds. Phytomedicine 19, 409–412. 10.1016/j.phymed.2011.10.005 22305279

[B43] Cogliati S. Clementi V. Francisco M. Crespo C. Argañaraz F. Grau R. (2020). Bacillus Subtilis Delays Neurodegeneration and Behavioral Impairment in the Alzheimer's Disease Model Caenorhabditis Elegans. J. Alzheimers Dis. 73, 1035–1052. 10.3233/JAD-190837 31884470

[B44] Crowe A. Henderson M. J. Anderson J. Titus S. A. Zakharov A. Simeonov A. (2020). Compound Screening in Cell-Based Models of Tau Inclusion Formation: Comparison of Primary Neuron and HEK293 Cell Assays. J. Biol. Chem. 295, 4001–4013. 10.1074/jbc.RA119.010532 32034092 PMC7086036

[B45] Cuevas E. Burks S. Raymick J. Robinson B. Gómez-Crisóstomo N. P. Escudero-Lourdes C. (2020). Tauroursodeoxycholic Acid (TUDCA) Is Neuroprotective in a Chronic Mouse Model of Parkinson's Disease. Nutr. Neurosci. 1, 1–18. 10.1080/1028415X.2020.1859729 33345721

[B46] Cui Q. Li X. Zhu H. (2016). Curcumin Ameliorates Dopaminergic Neuronal Oxidative Damage via Activation of the Akt/Nrf2 Pathway. Mol. Med. Rep. 13, 1381–1388. 10.3892/mmr.2015.4657 26648392

[B47] Cui W. Q. Qu Q. W. Wang J. P. Bai J. W. Bello-Onaghise G. Li Y. A. (2019). Discovery of Potential Anti-infective Therapy Targeting Glutamine Synthetase in Staphylococcus Xylosus. Front. Chem. 7, 381. 10.3389/fchem.2019.00381 31214565 PMC6558069

[B48] Dasagrandhi C. Park S. Jung W. K. Kim Y. M. (2018). Antibacterial and Biofilm Modulating Potential of Ferulic Acid-Grafted Chitosan against Human Pathogenic Bacteria. Int. J. Mol. Sci. 19. 10.3390/ijms19082157 PMC6121546 30042337

[B49] De Jesús-Cortés H. Xu P. Drawbridge J. Estill S. J. Huntington P. Tran S. (2012). Neuroprotective Efficacy of Aminopropyl Carbazoles in a Mouse Model of Parkinson Disease. Proc. Natl. Acad. Sci. U S A. 109, 17010–17015. 10.1073/pnas.1213956109 23027934 PMC3479520

[B50] De Oliveira D. M. P. Bohlmann L. Conroy T. Jen F. E. Everest-Dass A. Hansford K. A. (2020). Repurposing a Neurodegenerative Disease Drug to Treat Gram-Negative Antibiotic-Resistant Bacterial Sepsis. Sci. Transl Med. 12. 10.1126/scitranslmed.abb3791 33208501

[B51] Ding T. Li T. Wang Z. Li J. (2017). Curcumin Liposomes Interfere with Quorum Sensing System of Aeromonas Sobria and In Silico Analysis. Sci. Rep. 7, 8612. 10.1038/s41598-017-08986-9 28819178 PMC5561023

[B52] Diomede L. Cassata G. Fiordaliso F. Salio M. Ami D. Natalello A. (2010). Tetracycline and its Analogues Protect *Caenorhabditis elegans* from β Amyloid-Induced Toxicity by Targeting Oligomers. Neurobiol. Dis. 40, 424–431. 10.1016/j.nbd.2010.07.002 20637283

[B53] Dong G. Liu H. Yu X. Zhang X. Lu H. Zhou T. (2018). Antimicrobial and Anti-biofilm Activity of Tannic Acid against *Staphylococcus aureus* . Nat. Prod. Res. 32, 2225–2228. 10.1080/14786419.2017.1366485 28826250

[B54] Dostal V. Roberts C. M. Link C. D. (2010). Genetic Mechanisms of Coffee Extract protection in a *Caenorhabditis elegans* Model of β-amyloid Peptide Toxicity. Genetics 186, 857–866. 10.1534/genetics.110.120436 20805557 PMC2975290

[B55] Doub J. B. Heil E. L. Ntem-Mensah A. Neeley R. Ching P. R. (2020). Rifabutin Use in Staphylococcus Biofilm Infections: A Case Series. Antibiotics 9, 326. 10.3390/antibiotics9060326 PMC7345564 32545793

[B56] Dragicevic N. Smith A. Lin X. Yuan F. Copes N. Delic V. (2011). Green tea Epigallocatechin-3-Gallate (EGCG) and Other Flavonoids Reduce Alzheimer's Amyloid-Induced Mitochondrial Dysfunction. J. Alzheimers Dis. 26, 507–521. 10.3233/JAD-2011-101629 21694462

[B57] Ermolaeva M. A. Schumacher B. (2014). Insights from the Worm: the *C. elegans* Model for Innate Immunity. Semin. Immunol. 26, 303–309. 10.1016/j.smim.2014.04.005 24856329 PMC4248339

[B58] Eskelinen M. H. Kivipelto M. (2010). Caffeine as a Protective Factor in Dementia and Alzheimer's Disease. J. Alzheimers Dis. 20 Suppl 1, S167–S174. 10.3233/Jad-2010-1404 20182054

[B59] Evason K. Collins J. J. Huang C. Hughes S. Kornfeld K. (2008). Valproic Acid Extends *Caenorhabditis elegans* Lifespan. Aging Cell 7, 305–317. 10.1111/j.1474-9726.2008.00375.x 18248662 PMC6838649

[B60] Faber P. W. Voisine C. King D. C. Bates E. A. Hart A. C. (2002). Glutamine/proline-rich PQE-1 Proteins Protect *Caenorhabditis elegans* Neurons from Huntingtin Polyglutamine Neurotoxicity. Proc. Natl. Acad. Sci. U S A. 99, 17131–17136. 10.1073/pnas.262544899 12486229 PMC139281

[B61] Farr S. A. Roesler E. Niehoff M. L. Roby D. A. McKee A. Morley J. E. (2019). Metformin Improves Learning and Memory in the SAMP8 Mouse Model of Alzheimer's Disease. J. Alzheimers Dis. 68, 1699–1710. 10.3233/JAD-181240 30958364

[B62] Fasano A. Visanji N. P. Liu L. W. Lang A. E. Pfeiffer R. F. (2015). Gastrointestinal Dysfunction in Parkinson's Disease. Lancet Neurol. 14, 625–639. 10.1016/S1474-4422(15)00007-1 25987282

[B63] Fatouros C. Pir G. J. Biernat J. Koushika S. P. Mandelkow E. Mandelkow E. M. (2012). Inhibition of Tau Aggregation in a Novel *Caenorhabditis elegans* Model of Tauopathy Mitigates Proteotoxicity. Hum. Mol. Genet. 21, 3587–3603. 10.1093/hmg/dds190 22611162

[B64] Ferrante R. J. Ryu H. Kubilus J. K. D'Mello S. Sugars K. L. Lee J. (2004). Chemotherapy for the Brain: the Antitumor Antibiotic Mithramycin Prolongs Survival in a Mouse Model of Huntington's Disease. J. Neurosci. 24, 10335–10342. 10.1523/JNEUROSCI.2599-04.2004 15548647 PMC2577231

[B65] Friedland R. P. (2015). Mechanisms of Molecular Mimicry Involving the Microbiota in Neurodegeneration. J. Alzheimers Dis. 45, 349–362. 10.3233/JAD-142841 25589730

[B66] Fu R. H. Wang Y. C. Chen C. S. Tsai R. T. Liu S. P. Chang W. L. (2014). Acetylcorynoline Attenuates Dopaminergic Neuron Degeneration and α-synuclein Aggregation in Animal Models of Parkinson's Disease. Neuropharmacology 82, 108–120. 10.1016/j.neuropharm.2013.08.007 23973292

[B67] Fung T. C. Vuong H. E. Luna C. D. G. Pronovost G. N. Aleksandrova A. A. Riley N. G. (2019). Intestinal Serotonin and Fluoxetine Exposure Modulate Bacterial Colonization in the Gut. Nat. Microbiol. 4, 2064–2073. 10.1038/s41564-019-0540-4 31477894 PMC6879823

[B68] Gamir-Morralla A. Sacristán S. Medina M. Iglesias T. (2019). Effects of Thioflavin T and GSK-3 Inhibition on Lifespan and Motility in a *Caenorhabditis elegans* Model of Tauopathy. J. Alzheimers Dis. Rep. 3, 47–57. 10.3233/ADR-180087 30842997 PMC6400111

[B69] Garnaud C. Champleboux M. Maubon D. Govin M. J. (2016). Histone Deacetylases and Their Inhibition in Candida Species. Front. Microbiol. 7, 1238. 10.3389/fmicb.2016.01238 27547205 PMC4974301

[B70] Gilbert-Girard S. Savijoki K. Yli-Kauhaluoma J. Fallarero A. (2020). Screening of FDA-Approved Drugs Using a 384-Well Plate-Based Biofilm Platform: The Case of Fingolimod. Microorganisms 8. 10.3390/microorganisms8111834 PMC7700524 33233348

[B71] Gong T. Xiang Y. Saleh M. G. Gao F. Chen W. Edden R. A. E. (2018). Inhibitory Motor Dysfunction in Parkinson's Disease Subtypes. J. Magn. Reson. Imaging 47, 1610–1615. 10.1002/jmri.25865 28960581 PMC5871546

[B72] Goya M. E. Xue F. Sampedro-Torres-Quevedo C. Arnaouteli S. Riquelme-Dominguez L. Romanowski A. (2020). Probiotic Bacillus Subtilis Protects against α-Synuclein Aggregation in C. elegans. Cell Rep 30, 367–e7. e367. 10.1016/j.celrep.2019.12.078 31940482 PMC6963774

[B73] Grossi C. Francese S. Casini A. Rosi M. C. Luccarini I. Fiorentini A. (2009). Clioquinol Decreases Amyloid-Beta burden and Reduces Working Memory Impairment in a Transgenic Mouse Model of Alzheimer's Disease. J. Alzheimers Dis. 17, 423–440. 10.3233/JAD-2009-1063 19363260

[B74] Gu C. Hu Q. Wu J. Mu C. Ren H. Liu C. F. (2018). P7C3 Inhibits LPS-Induced Microglial Activation to Protect Dopaminergic Neurons against Inflammatory Factor-Induced Cell Death *In Vitro* and *In Vivo* . Front Cel Neurosci 12, 400. 10.3389/fncel.2018.00400 PMC6230654 30455635

[B75] Guan X.-N. Z. T. Zhang T. Yang T. Dong Z. Yang S. Lan L. (2022). Covalent Sortase A Inhibitor ML346 Prevents *Staphylococcus aureus* Infection of Galleria Mellonella. RSC Med. Chem. 13, 138–149. 10.1039/d1md00316j 35308030 PMC8864484

[B76] Guo L. Shokeen B. He X. Shi W. Lux R. (2017). Streptococcus Mutans SpaP Binds to RadD of Fusobacterium Nucleatum Ssp. Polymorphum. Mol. Oral Microbiol. 32, 355–364. 10.1111/omi.12177 27976528 PMC5472499

[B77] Guo X. Yuan J. Song X. Wang X. Sun Q. Tian J. (2020). Bacteria Metabolites from Peganum Harmala L. Polysaccharides Inhibits polyQ Aggregation through Proteasome-Mediated Protein Degradation in *C. elegans* . Int. J. Biol. Macromol 161, 681–691. 10.1016/j.ijbiomac.2020.06.091 32544588

[B78] Gutierrez-Zepeda A. Santell R. Wu Z. Brown M. Wu Y. Khan I. (2005). Soy Isoflavone Glycitein Protects against Beta Amyloid-Induced Toxicity and Oxidative Stress in Transgenic *Caenorhabditis elegans* . BMC Neurosci. 6, 54. 10.1186/1471-2202-6-54 16122394 PMC1215487

[B79] Halliday M. Radford H. Zents K. A. M. Molloy C. Moreno J. A. Verity N. C. (2017). Repurposed Drugs Targeting eIF2α-P-Mediated Translational Repression Prevent Neurodegeneration in Mice. Brain 140, 1768–1783. 10.1093/brain/awx074 28430857 PMC5445255

[B80] Hamamichi S. Rivas R. N. Knight A. L. Cao S. Caldwell K. A. Caldwell G. A. (2008). Hypothesis-based RNAi Screening Identifies Neuroprotective Genes in a Parkinson's Disease Model. Proc. Natl. Acad. Sci. U S A. 105, 728–733. 10.1073/pnas.0711018105 18182484 PMC2206604

[B81] Hasegawa S. Goto S. Tsuji H. Okuno T. Asahara T. Nomoto K. (2015). Intestinal Dysbiosis and Lowered Serum Lipopolysaccharide-Binding Protein in Parkinson's Disease. Plos One 10, e0142164. 10.1371/journal.pone.0142164 26539989 PMC4634857

[B82] Hassan W. M. Merin D. A. Fonte V. Link C. D. (2009). AIP-1 Ameliorates Beta-Amyloid Peptide Toxicity in a *Caenorhabditis elegans* Alzheimer's Disease Model. Hum. Mol. Genet. 18, 2739–2747. 10.1093/hmg/ddp209 19414486 PMC2706681

[B83] Hazan S. (2020). Rapid Improvement in Alzheimer's Disease Symptoms Following Fecal Microbiota Transplantation: a Case Report. J. Int. Med. Res. 48, 300060520925930. 10.1177/0300060520925930 32600151 PMC7328362

[B84] Himeno E. Ohyagi Y. Ma L. Nakamura N. Miyoshi K. Sakae N. (2011). Apomorphine Treatment in Alzheimer Mice Promoting Amyloid-β Degradation. Ann. Neurol. 69, 248–256. 10.1002/ana.22319 21387370

[B85] Hobley L. Li B. Wood J. L. Kim S. H. Naidoo J. Ferreira A. S. (2017). Spermidine Promotes Bacillus Subtilis Biofilm Formation by Activating Expression of the Matrix Regulator slrR. J. Biol. Chem. 292, 12041–12053. 10.1074/jbc.M117.789644 28546427 PMC5519356

[B86] Hosokawa M. Arai T. Masuda-Suzukake M. Nonaka T. Yamashita M. Akiyama H. (2012). Methylene Blue Reduced Abnormal Tau Accumulation in P301L Tau Transgenic Mice. Plos One 7, e52389. 10.1371/journal.pone.0052389 23285020 PMC3527507

[B87] Huang H. Xu H. Luo Q. He J. Li M. Chen H. (2019). Fecal Microbiota Transplantation to Treat Parkinson's Disease with Constipation: A Case Report. Medicine (Baltimore) 98, e16163. 10.1097/MD.0000000000016163 31261545 PMC6616439

[B88] Huang H. K. Wang J. H. Lei W. Y. Chen C. L. Chang C. Y. Liou L. S. (2018a). *Helicobacter pylori* Infection Is Associated with an Increased Risk of Parkinson's Disease: A Population-Based Retrospective Cohort Study. Parkinsonism Relat. Disord. 47, 26–31. 10.1016/j.parkreldis.2017.11.331 29174171

[B89] Huang M. Liang Y. Chen H. Xu B. Chai C. Xing P. (2018b). The Role of Fluoxetine in Activating Wnt/β-Catenin Signaling and Repressing β-Amyloid Production in an Alzheimer Mouse Model. Front. Aging Neurosci. 10, 164. 10.3389/fnagi.2018.00164 29910725 PMC5992518

[B90] Inden M. Kitamura Y. Abe M. Tamaki A. Takata K. Taniguchi T. (2011). Parkinsonian Rotenone Mouse Model: Reevaluation of Long-Term Administration of Rotenone in C57BL/6 Mice. Biol. Pharm. Bull. 34, 92–96. 10.1248/bpb.34.92 21212524

[B91] Jellinger K. A. (2010). Basic Mechanisms of Neurodegeneration: a Critical Update. J. Cel Mol Med 14, 457–487. 10.1111/j.1582-4934.2010.01010.x PMC3823450 20070435

[B92] Junker L. M. Clardy J. (2007). High-throughput Screens for Small-Molecule Inhibitors of *Pseudomonas aeruginosa* Biofilm Development. Antimicrob. Agents Chemother. 51, 3582–3590. 10.1128/AAC.00506-07 17664319 PMC2043262

[B93] Kaizaki A. Tien L. T. Pang Y. Cai Z. Tanaka S. Numazawa S. (2013). Celecoxib Reduces Brain Dopaminergic Neuronaldysfunction, and Improves Sensorimotor Behavioral Performance in Neonatal Rats Exposed to Systemic Lipopolysaccharide. J. Neuroinflammation 10, 45. 10.1186/1742-2094-10-45 23561827 PMC3637465

[B94] Kali A. Bhuvaneshwar D. Charles P. M. Seetha K. S. (2016). Antibacterial Synergy of Curcumin with Antibiotics against Biofilm Producing Clinical Bacterial Isolates. J. Basic Clin. Pharm. 7, 93–96. 10.4103/0976-0105.183265 27330262 PMC4910474

[B95] Kautu B. B. Carrasquilla A. Hicks M. L. Caldwell K. A. Caldwell G. A. (2013). Valproic Acid Ameliorates *C. elegans* Dopaminergic Neurodegeneration with Implications for ERK-MAPK Signaling. Neurosci. Lett. 541, 116–119. 10.1016/j.neulet.2013.02.026 23485787 PMC3655695

[B96] Keowkase R. Aboukhatwa M. Adam B. L. Beach J. W. Terry A. V. Jr. Buccafussco J. J. (2010a). Neuroprotective Effects and Mechanism of Cognitive-Enhancing Choline Analogs JWB 1-84-1 and JAY 2-22-33 in Neuronal Culture and *Caenorhabditis elegans* . Mol. Neurodegener 5, 59. 10.1186/1750-1326-5-59 21162742 PMC3017027

[B97] Keowkase R. Aboukhatwa M. Luo Y. (2010b). Fluoxetine Protects against Amyloid-Beta Toxicity, in Part via Daf-16 Mediated Cell Signaling Pathway, in *Caenorhabditis elegans* . Neuropharmacology 59, 358–365. 10.1016/j.neuropharm.2010.04.008 20420844 PMC2926186

[B98] Kidd S. K. Schneider J. S. (2011). Protective Effects of Valproic Acid on the Nigrostriatal Dopamine System in a 1-Methyl-4-Phenyl-1,2,3,6-Tetrahydropyridine Mouse Model of Parkinson's Disease. Neuroscience 194, 189–194. 10.1016/j.neuroscience.2011.08.010 21846494 PMC3196607

[B99] Koh S. H. Lee S. M. Kim H. Y. Lee K. Y. Lee Y. J. Kim H. T. (2006). The Effect of Epigallocatechin Gallate on Suppressing Disease Progression of ALS Model Mice. Neurosci. Lett. 395, 103–107. 10.1016/j.neulet.2005.10.056 16356650

[B100] Kosuru R. Y. Roy A. Bera S. (2021). Antagonistic Roles of Gallates and Ascorbic Acid in Pyomelanin Biosynthesis of *Pseudomonas aeruginosa* Biofilms. Curr. Microbiol. 78, 3843–3852. 10.1007/s00284-021-02655-x 34554299

[B101] Koutzoumis D. N. Vergara M. Pino J. Buddendorff J. Khoshbouei H. Mandel R. J. (2020). Alterations of the Gut Microbiota with Antibiotics Protects Dopamine Neuron Loss and Improve Motor Deficits in a Pharmacological Rodent Model of Parkinson's Disease. Exp. Neurol. 325, 113159. 10.1016/j.expneurol.2019.113159 31843492

[B102] Kurniawan A. Yamamoto T. (2013). Biofilm Polymer for Biosorption of Pollutant Ions. Proced. Environ. Sci. 17, 179–187. 10.1016/j.proenv.2013.02.027

[B103] Kuwahara T. Koyama A. Koyama S. Yoshina S. Ren C. H. Kato T. (2008). A Systematic RNAi Screen Reveals Involvement of Endocytic Pathway in Neuronal Dysfunction in Alpha-Synuclein Transgenic *C. elegans* . Hum. Mol. Genet. 17, 2997–3009. 10.1093/hmg/ddn198 18617532

[B104] Lalouckova K. Mala L. Marsik P. Skrivanova E. (2021). *In Vitro* Antibacterial Effect of the Methanolic Extract of the Korean Soybean Fermented Product Doenjang against *Staphylococcus aureus* . Animals 11, 2319. 10.3390/ani11082319 34438775 PMC8388408

[B105] Laloux C. Derambure P. Houdayer E. Jacquesson J. M. Bordet R. Destée A. (2008). Effect of Dopaminergic Substances on Sleep/wakefulness in saline- and MPTP-Treated Mice. J. Sleep Res. 17, 101–110. 10.1111/j.1365-2869.2008.00625.x 18275560

[B106] Lee J. H. Kim Y. G. Ryu S. Y. Cho M. H. Lee J. (2014). Ginkgolic Acids and Ginkgo Biloba Extract Inhibit *Escherichia coli* O157:H7 and *Staphylococcus aureus* Biofilm Formation. Int. J. Food Microbiol. 174, 47–55. 10.1016/j.ijfoodmicro.2013.12.030 24457153

[B107] Lehtonen Š. Jaronen M. Vehviläinen P. Lakso M. Rudgalvyte M. Keksa-Goldsteine V. (2016). Inhibition of Excessive Oxidative Protein Folding Is Protective in MPP(+) Toxicity-Induced Parkinson's Disease Models. Antioxid. Redox Signal. 25, 485–497. 10.1089/ars.2015.6402 27139804

[B108] Li C. Cui L. Yang Y. Miao J. Zhao X. Zhang J. (2019a). Gut Microbiota Differs between Parkinson's Disease Patients and Healthy Controls in Northeast China. Front. Mol. Neurosci. 12, 171. 10.3389/fnmol.2019.00171 31354427 PMC6637281

[B109] Li F. Zhang Y. Lu X. Shi J. Gong Q. (2019b). Icariin Improves the Cognitive Function of APP/PS1 Mice via Suppressing Endoplasmic Reticulum Stress. Life Sci. 234, 116739. 10.1016/j.lfs.2019.116739 31400352

[B110] Liachko N. F. McMillan P. J. Guthrie C. R. Bird T. D. Leverenz J. B. Kraemer B. C. (2013). CDC7 Inhibition Blocks Pathological TDP-43 Phosphorylation and Neurodegeneration. Ann. Neurol. 74, 39–52. 10.1002/ana.23870 23424178 PMC3775949

[B111] Lim G. P. Chu T. Yang F. Beech W. Frautschy S. A. Cole G. M. (2001). The Curry Spice Curcumin Reduces Oxidative Damage and Amyloid Pathology in an Alzheimer Transgenic Mouse. J. Neurosci. 21, 8370–8377. 10.1523/jneurosci.21-21-08370.2001 11606625 PMC6762797

[B112] Lim K. H. (2019). Diverse Misfolded Conformational Strains and Cross-Seeding of Misfolded Proteins Implicated in Neurodegenerative Diseases. Front. Mol. Neurosci. 12, 158. 10.3389/fnmol.2019.00158 31338019 PMC6629833

[B113] Link C. D. (1995). Expression of Human Beta-Amyloid Peptide in Transgenic *Caenorhabditis elegans* . Proc. Natl. Acad. Sci. U S A. 92, 9368–9372. 10.1073/pnas.92.20.9368 7568134 PMC40986

[B114] Link C. D. Taft A. Kapulkin V. Duke K. Kim S. Fei Q. (2003). Gene Expression Analysis in a Transgenic *Caenorhabditis elegans* Alzheimer's Disease Model. Neurobiol. Aging 24, 397–413. 10.1016/s0197-4580(02)00224-5 12600716

[B115] Liu Z. Hamamichi S. Lee B. D. Yang D. Ray A. Caldwell G. A. (2011). Inhibitors of LRRK2 Kinase Attenuate Neurodegeneration and Parkinson-like Phenotypes in *Caenorhabditis elegans* and Drosophila Parkinson's Disease Models. Hum. Mol. Genet. 20, 3933–3942. 10.1093/hmg/ddr312 21768216 PMC3177653

[B116] Locke C. J. Fox S. A. Caldwell G. A. Caldwell K. A. (2008). Acetaminophen Attenuates Dopamine Neuron Degeneration in Animal Models of Parkinson's Disease. Neurosci. Lett. 439, 129–133. 10.1016/j.neulet.2008.05.003 18514411

[B117] Lublin A. Isoda F. Patel H. Yen K. Nguyen L. Hajje D. (2011). FDA-approved Drugs that Protect Mammalian Neurons from Glucose Toxicity Slow Aging Dependent on Cbp and Protect against Proteotoxicity. Plos One 6, ARTN e27762. 10.1371/journal.pone.0027762 PMC3218048 22114686

[B118] Luo J. Dong B. Wang K. Cai S. Liu T. Cheng X. (2017). Baicalin Inhibits Biofilm Formation, Attenuates the Quorum Sensing-Controlled Virulence and Enhances *Pseudomonas aeruginosa* Clearance in a Mouse Peritoneal Implant Infection Model. Plos One 12, e0176883. 10.1371/journal.pone.0176883 28453568 PMC5409170

[B119] Ma J. Wang R. Chen T. Jiang S. Xu A. (2021). Protective Effects of Baicalin in a *Caenorhabditis elegans* Model of Parkinson's Disease. Toxicol. Res. (Camb) 10, 409–417. 10.1093/toxres/tfaa107 34141154 PMC8201589

[B120] Martin L. J. (2012). Biology of Mitochondria in Neurodegenerative Diseases. Prog. Mol. Biol. Transl Sci. 107, 355–415. 10.1016/B978-0-12-385883-2.00005-9 22482456 PMC3530202

[B121] Martinez B. A. Kim H. Ray A. Caldwell G. A. Caldwell K. A. (2015). A Bacterial Metabolite Induces Glutathione-Tractable Proteostatic Damage, Proteasomal Disturbances, and PINK1-dependent Autophagy in *C. elegans* . Cell Death Dis 6, e1908. 10.1038/cddis.2015.270 26469957 PMC4632299

[B122] Matlack K. E. S. Tardiff D. F. Narayan P. Hamamichi S. Caldwell K. A. Caldwell G. A. (2014). Clioquinol Promotes the Degradation of Metal-dependent Amyloid-β (Aβ) Oligomers to Restore Endocytosis and Ameliorate Aβ Toxicity. Proc. Natl. Acad. Sci. U.S.A. 111, 4013–4018. 10.1073/pnas.1402228111 24591589 PMC3964050

[B123] McColl G. Roberts B. R. Pukala T. L. Kenche V. B. Roberts C. M. Link C. D. (2012). Utility of an Improved Model of Amyloid-Beta (Aβ₁₋₄₂) Toxicity in *Caenorhabditis elegans* for Drug Screening for Alzheimer's Disease. Mol. Neurodegener 7, 57. 10.1186/1750-1326-7-57 23171715 PMC3519830

[B124] McCormick A. V. Wheeler J. M. Guthrie C. R. Liachko N. F. Kraemer B. C. (2013). Dopamine D2 Receptor Antagonism Suppresses Tau Aggregation and Neurotoxicity. Biol. Psychiatry 73, 464–471. 10.1016/j.biopsych.2012.08.027 23140663 PMC3570611

[B125] Meier S. Bell M. Lyons D. N. Ingram A. Chen J. Gensel J. C. (2015). Identification of Novel Tau Interactions with Endoplasmic Reticulum Proteins in Alzheimer's Disease Brain. J. Alzheimers Dis. 48, 687–702. 10.3233/JAD-150298 26402096 PMC4881838

[B126] Memariani H. Memariani M. Ghasemian A. (2019). An Overview on Anti-biofilm Properties of Quercetin against Bacterial Pathogens. World J. Microbiol. Biotechnol. 35, 143. 10.1007/s11274-019-2719-5 31493142

[B127] Miyasaka T. Ding Z. Gengyo-Ando K. Oue M. Yamaguchi H. Mitani S. (2005). Progressive Neurodegeneration in *C. elegans* Model of Tauopathy. Neurobiol. Dis. 20, 372–383. 10.1016/j.nbd.2005.03.017 16242642

[B128] Miyasaka T. Xie C. Yoshimura S. Shinzaki Y. Yoshina S. Kage-Nakadai E. (2016). Curcumin Improves Tau-Induced Neuronal Dysfunction of Nematodes. Neurobiol. Aging 39, 69–81. 10.1016/j.neurobiolaging.2015.11.004 26923403

[B129] Mocko J. B. Kern A. Moosmann B. Behl C. Hajieva P. (2010). Phenothiazines Interfere with Dopaminergic Neurodegeneration in *Caenorhabditis elegans* Models of Parkinson's Disease. Neurobiol. Dis. 40, 120–129. 10.1016/j.nbd.2010.03.019 20403440

[B130] Mori T. Rezai-Zadeh K. Koyama N. Arendash G. W. Yamaguchi H. Kakuda N. (2012). Tannic Acid Is a Natural β-secretase Inhibitor that Prevents Cognitive Impairment and Mitigates Alzheimer-like Pathology in Transgenic Mice. J. Biol. Chem. 287, 6912–6927. 10.1074/jbc.M111.294025 22219198 PMC3307267

[B131] Nishiwaki H. Ito M. Ishida T. Hamaguchi T. Maeda T. Kashihara K. (2020). Meta-Analysis of Gut Dysbiosis in Parkinson's Disease. Mov Disord. 35, 1626–1635. 10.1002/mds.28119 32557853

[B132] Ogawa N. Tanaka K. Asanuma M. Kawai M. Masumizu T. Kohno M. (1994). Bromocriptine Protects Mice against 6-hydroxydopamine and Scavenges Hydroxyl Free Radicals *In Vitro* . Brain Res. 657, 207–213. 10.1016/0006-8993(94)90969-5 7820619

[B133] Opoku-Temeng C. Miller N. J. Sintim H. O. (2017). Hydroxybenzylidene-indolinones, C-Di-AMP Synthase Inhibitors, Have Antibacterial and Anti-biofilm Activities and Also Re-sensitize Resistant Bacteria to Methicillin and Vancomycin. RSC Adv. 7, 8288–8294. 10.1039/c6ra28443d

[B134] Park S. H. Lee J. H. Shin J. Kim J. S. Cha B. Lee S. (2021). Cognitive Function Improvement after Fecal Microbiota Transplantation in Alzheimer's Dementia Patient: a Case Report. Curr. Med. Res. Opin. 37, 1739–1744. 10.1080/03007995.2021.1957807 34289768

[B135] Patil S. P. Jain P. D. Ghumatkar P. J. Tambe R. Sathaye S. (2014). Neuroprotective Effect of Metformin in MPTP-Induced Parkinson's Disease in Mice. Neuroscience 277, 747–754. 10.1016/j.neuroscience.2014.07.046 25108167

[B136] Paytubi S. de La Cruz M. Tormo J. R. Martín J. González I. González-Menendez V. (2017). A High-Throughput Screening Platform of Microbial Natural Products for the Discovery of Molecules with Antibiofilm Properties against Salmonella. Front. Microbiol. 8, 326. 10.3389/fmicb.2017.00326 28303128 PMC5332434

[B137] Pelling H. Nzakizwanayo J. Milo S. Denham E. L. MacFarlane W. M. Bock L. J. (2019). Bacterial Biofilm Formation on Indwelling Urethral Catheters. Lett. Appl. Microbiol. 68, 277–293. 10.1111/lam.13144 30811615

[B138] Peterson C. T. (2020). Dysfunction of the Microbiota-Gut-Brain Axis in Neurodegenerative Disease: The Promise of Therapeutic Modulation with Prebiotics, Medicinal Herbs, Probiotics, and Synbiotics. J. Evid. Based Integr. Med. 25, 2515690X20957225. 10.1177/2515690X20957225 PMC7586271 33092396

[B139] Pham J. V. Yilma M. A. Feliz A. Majid M. T. Maffetone N. Walker J. R. (2019). A Review of the Microbial Production of Bioactive Natural Products and Biologics. Front. Microbiol. 10, 1404. 10.3389/fmicb.2019.01404 31281299 PMC6596283

[B140] Ramsey C. P. Glass C. A. Montgomery M. B. Lindl K. A. Ritson G. P. Chia L. A. (2007). Expression of Nrf2 in Neurodegenerative Diseases. J. Neuropathol. Exp. Neurol. 66, 75–85. 10.1097/nen.0b013e31802d6da9 17204939 PMC2253896

[B141] Ray A. Martinez B. A. Berkowitz L. A. Caldwell G. A. Caldwell K. A. (2014a). Mitochondrial Dysfunction, Oxidative Stress, and Neurodegeneration Elicited by a Bacterial Metabolite in a *C. elegans* Parkinson's Model. Cel Death Dis 5, e984. 10.1038/cddis.2013.513 PMC4040705 24407237

[B142] Ray A. Zhang S. Rentas C. Caldwell K. A. Caldwell G. A. (2014b). RTCB-1 Mediates Neuroprotection via XBP-1 mRNA Splicing in the Unfolded Protein Response Pathway. J. Neurosci. 34, 16076–16085. 10.1523/JNEUROSCI.1945-14.2014 25429148 PMC4244473

[B143] Regitz C. Dussling L. M. Wenzel U. (2014). Amyloid-beta (Aβ₁₋₄₂)-Induced Paralysis in *Caenorhabditis elegans* Is Inhibited by the Polyphenol Quercetin through Activation of Protein Degradation Pathways. Mol. Nutr. Food Res. 58, 1931–1940. 10.1002/mnfr.201400014 25066301

[B144] Ren H. Zhai W. Lu X. Wang G. (2021). The Cross-Links of Endoplasmic Reticulum Stress, Autophagy, and Neurodegeneration in Parkinson's Disease. Front. Aging Neurosci. 13, 691881. 10.3389/fnagi.2021.691881 34168552 PMC8218021

[B145] Rojas F. Gonzalez D. Cortes N. Ampuero E. Hernández D. E. Fritz E. (2015). Reactive Oxygen Species Trigger Motoneuron Death in Non-cell-autonomous Models of ALS through Activation of C-Abl Signaling. Front. Cel Neurosci 9, 203. 10.3389/fncel.2015.00203 PMC4460879 26106294

[B146] Rojo A. I. Pajares M. García-Yagüe A. J. Buendia I. Van Leuven F. Yamamoto M. (2018). Deficiency in the Transcription Factor NRF2 Worsens Inflammatory Parameters in a Mouse Model with Combined Tauopathy and Amyloidopathy. Redox Biol. 18, 173–180. 10.1016/j.redox.2018.07.006 30029164 PMC6052199

[B147] Saewanee N. Praputpittaya T. Malaiwong N. Chalorak P. Meemon K. (2021). Neuroprotective Effect of Metformin on Dopaminergic Neurodegeneration and α-synuclein Aggregation in *C. elegans* Model of Parkinson's Disease. Neurosci. Res. 162, 13–21. 10.1016/j.neures.2019.12.017 31881233

[B148] Sampson T. R. Challis C. Jain N. Moiseyenko A. Ladinsky M. S. Shastri G. G. (2020). A Gut Bacterial Amyloid Promotes α-synuclein Aggregation and Motor Impairment in Mice. Elife 9. 10.7554/eLife.53111 PMC7012599 32043464

[B149] Sampson T. R. Debelius J. W. Thron T. Janssen S. Shastri G. G. Ilhan Z. E. (2016). Gut Microbiota Regulate Motor Deficits and Neuroinflammation in a Model of Parkinson's Disease. Cell 167, 1469–e12. 10.1016/j.cell.2016.11.018 27912057 PMC5718049

[B150] Sanchis A. García-Gimeno M. A. Cañada-Martínez A. J. Sequedo M. D. Millán J. M. Sanz P. (2019). Metformin Treatment Reduces Motor and Neuropsychiatric Phenotypes in the zQ175 Mouse Model of Huntington Disease. Exp. Mol. Med. 51, 1–16. 10.1038/s12276-019-0264-9 PMC6549163 31165723

[B151] Sandlie I. Solberg K. Kleppe K. (1980). The Effect of Caffeine on Cell Growth and Metabolism of Thymidine in *Escherichia coli* . Mutat. Res. 73, 29–41. 10.1016/0027-5107(80)90133-5 7019679

[B152] Sarkar S. Raymick J. Ray B. Lahiri D. K. Paule M. G. Schmued L. (2015). Oral Administration of Thioflavin T Prevents Beta Amyloid Plaque Formation in Double Transgenic AD Mice. Curr. Alzheimer Res. 12, 837–846. 10.2174/156720501209151019105647 26510980

[B153] Saxena S. Cabuy E. Caroni P. (2009). A Role for Motoneuron Subtype-Selective ER Stress in Disease Manifestations of FALS Mice. Nat. Neurosci. 12, 627–636. 10.1038/nn.2297 19330001

[B154] Sedjahtera A. Gunawan L. Bray L. Hung L. W. Parsons J. Okamura N. (2018). Targeting Metals Rescues the Phenotype in an Animal Model of Tauopathy. Metallomics 10, 1339–1347. 10.1039/c8mt00153g 30168573

[B155] Serra D. O. Mika F. Richter A. M. Hengge R. (2016). The green tea Polyphenol EGCG Inhibits *E. coli* Biofilm Formation by Impairing Amyloid Curli Fibre Assembly and Downregulating the Biofilm Regulator CsgD via the σ(E) -dependent sRNA RybB. Mol. Microbiol. 101, 136–151. 10.1111/mmi.13379 26992034

[B156] Shaw J. D. Brodke D. S. Williams D. L. Ashton N. N. (2020). Methylene Blue Is an Effective Disclosing Agent for Identifying Bacterial Biofilms on Orthopaedic Implants. J. Bone Jt. Surg Am 102, 1784–1791. 10.2106/JBJS.20.00091 33086345

[B157] Siddiqui M. F. Winters H. Maqbool F. Qayyum S. Singh L. Ullah I. (2019). Tannic Acid Treatment to Deter Microbial Biofouling in Flow Cell System and on RO Membrane in Drip Flow Reactor. Dwt 171, 62–66. 10.5004/dwt.2019.24767

[B158] Siles S. A. Srinivasan A. Pierce C. G. Lopez-Ribot J. L. Ramasubramanian A. K. (2013). High-throughput Screening of a Collection of Known Pharmacologically Active Small Compounds for Identification of Candida Albicans Biofilm Inhibitors. Antimicrob. Agents Chemother. 57, 3681–3687. 10.1128/AAC.00680-13 23689719 PMC3719724

[B159] Singh D. Gupta S. Verma I. Morsy M. A. Nair A. B. Ahmed A. F. (2021). Hidden Pharmacological Activities of Valproic Acid: A New Insight. Biomed. Pharmacother. 142, 112021. 10.1016/j.biopha.2021.112021 34463268

[B160] Singh N. A. Mandal A. K. Khan Z. A. (2016). Potential Neuroprotective Properties of Epigallocatechin-3-Gallate (EGCG). Nutr. J. 15, 60. 10.1186/s12937-016-0179-4 27268025 PMC4897892

[B161] Sood A. Warren Beach J. Webster S. J. Terry A. V. Buccafusco J. J. (2007). The Effects of JWB1-84-1 on Memory-Related Task Performance by Amyloid Abeta Transgenic Mice and by Young and Aged Monkeys. Neuropharmacology 53, 588–600. 10.1016/j.neuropharm.2007.06.028 17698153

[B162] Stone G. Wood P. Dixon L. Keyhan M. Matin A. (2002). Tetracycline Rapidly Reaches All the Constituent Cells of Uropathogenic *Escherichia coli* Biofilms. Antimicrob. Agents Chemother. 46, 2458–2461. 10.1128/AAC.46.8.2458-2461.2002 12121918 PMC127323

[B163] Sun J. Xu J. Ling Y. Wang F. Gong T. Yang C. (2019). Fecal Microbiota Transplantation Alleviated Alzheimer's Disease-like Pathogenesis in APP/PS1 Transgenic Mice. Transl Psychiatry 9, 189. 10.1038/s41398-019-0525-3 31383855 PMC6683152

[B164] Suo H. Wang P. Tong J. Cai L. Liu J. Huang D. (2015). NRSF Is an Essential Mediator for the Neuroprotection of Trichostatin A in the MPTP Mouse Model of Parkinson's Disease. Neuropharmacology 99, 67–78. 10.1016/j.neuropharm.2015.07.015 26188143

[B165] Suresh D. Sabir S. Yu T. T. Wenholz D. Das T. Black D. S. (2021). Natural Product Rottlerin Derivatives Targeting Quorum Sensing. Molecules 26. 10.3390/molecules26123745 PMC8235494 34205355

[B166] Takahashi H. Kashimura M. Koiso H. Kuda T. Kimura B. (2013). Use of Ferulic Acid as a Novel Candidate of Growth Inhibiting Agent against Listeria Monocytogenes in Ready-To-Eat Food. Food Control 33, 244–248. 10.1016/j.foodcont.2013.03.013

[B167] Tan A. H. Mahadeva S. Marras C. Thalha A. M. Kiew C. K. Yeat C. M. (2015). *Helicobacter pylori* Infection Is Associated with Worse Severity of Parkinson's Disease. Parkinsonism Relat. Disord. 21, 221–225. 10.1016/j.parkreldis.2014.12.009 25560322

[B168] Tauffenberger A. Julien C. Parker J. A. (2013). Evaluation of Longevity Enhancing Compounds against Transactive Response DNA-Binding Protein-43 Neuronal Toxicity. Neurobiol. Aging 34, 2175–2182. 10.1016/j.neurobiolaging.2013.03.014 23591130

[B169] Tchantchou F. Xu Y. Wu Y. Christen Y. Luo Y. (2007). EGb 761 Enhances Adult Hippocampal Neurogenesis and Phosphorylation of CREB in Transgenic Mouse Model of Alzheimer's Disease. FASEB J. 21, 2400–2408. 10.1096/fj.06-7649com 17356006

[B170] Therrien M. Parker J. A. (2014). Worming Forward: Amyotrophic Lateral Sclerosis Toxicity Mechanisms and Genetic Interactions in *Caenorhabditis elegans* . Front. Genet. 5, 85. 10.3389/fgene.2014.00085 24860590 PMC4029022

[B171] Thongbhubate K. Nakafuji Y. Matsuoka R. Kakegawa S. Suzuki H. (2021). Effect of Spermidine on Biofilm Formation in *Escherichia coli* K-12. J. Bacteriol. 203. 10.1128/JB.00652-20 PMC8088603 33685971

[B172] Tsai C. W. Tsai R. T. Liu S. P. Chen C. S. Tsai M. C. Chien S. H. (2017). Neuroprotective Effects of Betulin in Pharmacological and Transgenic *Caenorhabditis elegans* Models of Parkinson's Disease. Cel Transpl. 26, 1903–1918. 10.1177/0963689717738785 PMC5802634 29390878

[B173] Tzeng S. R. Huang Y. W. Zhang Y. Q. Yang C. Y. Chien H. S. Chen Y. R. (2020). A Celecoxib Derivative Eradicates Antibiotic-Resistant *Staphylococcus aureus* and Biofilms by Targeting YidC2 Translocase. Int. J. Mol. Sci. 21. 10.3390/ijms21239312 PMC7730571 33297331

[B174] Umeda T. Tanaka A. Sakai A. Yamamoto A. Sakane T. Tomiyama T. (2018). Intranasal Rifampicin for Alzheimer's Disease Prevention. Alzheimers Dement (N Y) 4, 304–313. 10.1016/j.trci.2018.06.012 30094330 PMC6076366

[B175] Urrutia A. García-Angulo V. A. Fuentes A. Caneo M. Legüe M. Urquiza S. (2020). Bacterially Produced Metabolites Protect *C. elegans* Neurons from Degeneration. Plos Biol. 18, e3000638. 10.1371/journal.pbio.3000638 32208418 PMC7092960

[B176] Vaccaro A. Patten S. A. Aggad D. Julien C. Maios C. Kabashi E. (2013). Pharmacological Reduction of ER Stress Protects against TDP-43 Neuronal Toxicity *In Vivo* . Neurobiol. Dis. 55, 64–75. 10.1016/j.nbd.2013.03.015 23567652

[B177] Vaccaro A. Patten S. A. Ciura S. Maios C. Therrien M. Drapeau P. (2012a). Methylene Blue Protects against TDP-43 and FUS Neuronal Toxicity in *C. elegans* and *D. rerio* . Plos One 7, e42117. 10.1371/journal.pone.0042117 22848727 PMC3407135

[B178] Vaccaro A. Tauffenberger A. Ash P. E. Carlomagno Y. Petrucelli L. Parker J. A. (2012b). TDP-1/TDP-43 Regulates Stress Signaling and Age-dependent Proteotoxicity in *Caenorhabditis elegans* . Plos Genet. 8, e1002806. 10.1371/journal.pgen.1002806 22792076 PMC3390363

[B179] Vajjala A. Biswas D. Tay W. H. Hanski E. Kline K. A. (2019). Streptolysin-induced Endoplasmic Reticulum Stress Promotes Group A Streptococcal Host-Associated Biofilm Formation and Necrotising Fasciitis. Cell Microbiol 21, e12956. 10.1111/cmi.12956 30239106

[B180] Van Pelt K. M. Truttmann M. C. (2020). *Caenorhabditis elegans* as a Model System for Studying Aging-Associated Neurodegenerative Diseases. Transl Med. Aging 4, 60–72. 10.1016/j.tma.2020.05.001 34327290 PMC8317484

[B181] Varma H. Cheng R. Voisine C. Hart A. C. Stockwell B. R. (2007). Inhibitors of Metabolism rescue Cell Death in Huntington's Disease Models. Proc. Natl. Acad. Sci. U S A. 104, 14525–14530. 10.1073/pnas.0704482104 17726098 PMC1964858

[B182] Vaughn A. R. Haas K. N. Burney W. Andersen E. Clark A. K. Crawford R. (2017). Potential Role of Curcumin against Biofilm-Producing Organisms on the Skin: A Review. Phytother Res. 31, 1807–1816. 10.1002/ptr.5912 28884496

[B183] Ved R. Saha S. Westlund B. Perier C. Burnam L. Sluder A. (2005). Similar Patterns of Mitochondrial Vulnerability and rescue Induced by Genetic Modification of Alpha-Synuclein, Parkin, and DJ-1 in *Caenorhabditis elegans* . J. Biol. Chem. 280, 42655–42668. 10.1074/jbc.M505910200 16239214 PMC3910375

[B184] Verma R. Gurumurthy M. Yeo B. C. M. Lu Q. Naftalin C. M. Paton N. I. (2022). Effects of Increasing Concentrations of Rifampicin on Different *Mycobacterium tuberculosis* Lineages in a Whole-Blood Bactericidal Activity Assay. Antimicrob. Agents Chemother. 66, AAC0169921. 10.1128/AAC.01699-21 PMC8846316 34871090

[B185] Vieira F. G. Ping Q. Moreno A. J. Kidd J. D. Thompson K. Jiang B. (2015). Guanabenz Treatment Accelerates Disease in a Mutant SOD1 Mouse Model of ALS. Plos One 10, e0135570. 10.1371/journal.pone.0135570 26288094 PMC4545826

[B186] Viszwapriya D. Subramenium G. A. Prithika U. Balamurugan K. Pandian S. K. (2016). Betulin Inhibits Virulence and Biofilm of Streptococcus Pyogenes by Suppressing ropB Core Regulon, sagA and dltA. Pathog. Dis. 74. 10.1093/femspd/ftw088 27596811

[B187] Voisine C. Varma H. Walker N. Bates E. A. Stockwell B. R. Hart A. C. (2007). Identification of Potential Therapeutic Drugs for huntington's Disease Using *Caenorhabditis elegans* . Plos One 2, e504. 10.1371/journal.pone.0000504 17551584 PMC1876812

[B188] Wang C. Saar V. Leung K. L. Chen L. Wong G. (2018). Human Amyloid β Peptide and Tau Co-expression Impairs Behavior and Causes Specific Gene Expression Changes in *Caenorhabditis elegans* . Neurobiol. Dis. 109, 88–101. 10.1016/j.nbd.2017.10.003 28982592

[B189] Wang C. Lau C. Y. Ma F. Zheng C. (2021a). Genome-wide Screen Identifies Curli Amyloid Fibril as a Bacterial Component Promoting Host Neurodegeneration. Proc. Natl. Acad. Sci. U.S.A. 118. 10.1073/pnas.2106504118 PMC8403922 34413194

[B190] Wang H. Liu X. Tan C. Zhou W. Jiang J. Peng W. (2020a). Bacterial, Viral, and Fungal Infection-Related Risk of Parkinson's Disease: Meta-Analysis of Cohort and Case-Control Studies. Brain Behav. 10, e01549. 10.1002/brb3.1549 32017453 PMC7066372

[B191] Wang I. F. Guo B. S. Liu Y. C. Wu C. C. Yang C. H. Tsai K. J. (2012). Autophagy Activators rescue and Alleviate Pathogenesis of a Mouse Model with Proteinopathies of the TAR DNA-Binding Protein 43. Proc. Natl. Acad. Sci. U S A. 109, 15024–15029. 10.1073/pnas.1206362109 22932872 PMC3443184

[B192] Wang J. Farr G. W. Hall D. H. Li F. Furtak K. Dreier L. (2009). An ALS-Linked Mutant SOD1 Produces a Locomotor Defect Associated with Aggregation and Synaptic Dysfunction when Expressed in Neurons of *Caenorhabditis elegans* . Plos Genet. 5, e1000350. 10.1371/journal.pgen.1000350 19165329 PMC2621352

[B193] Wang N. Zhou Y. Zhao L. Wang C. Ma W. Ge G. (2020b). Ferulic Acid Delayed Amyloid β-induced Pathological Symptoms by Autophagy Pathway via a Fasting-like Effect in *Caenorhabditis elegans* . Food Chem. Toxicol. 146, 111808. 10.1016/j.fct.2020.111808 33045309

[B194] Wang N. Y. Li J. N. Liu W. L. Huang Q. Li W. X. Tan Y. H. (2021b). Ferulic Acid Ameliorates Alzheimer's Disease-like Pathology and Repairs Cognitive Decline by Preventing Capillary Hypofunction in APP/PS1 Mice. Neurotherapeutics 18, 1064–1080. 10.1007/s13311-021-01024-7 33786807 PMC8423929

[B195] Watson E. MacNeil L. T. Ritter A. D. Yilmaz L. S. Rosebrock A. P. Caudy A. A. (2014). Interspecies Systems Biology Uncovers Metabolites Affecting *C. elegans* Gene Expression and Life History Traits. Cell 156, 1336–1337. 10.1016/j.cell.2014.02.036 24529378 PMC4169190

[B196] Williamson T. P. Johnson D. A. Johnson J. A. (2012). Activation of the Nrf2-ARE Pathway by siRNA Knockdown of Keap1 Reduces Oxidative Stress and Provides Partial protection from MPTP-Mediated Neurotoxicity. Neurotoxicology 33, 272–279. 10.1016/j.neuro.2012.01.015 22342405 PMC3521526

[B197] Wong S. Q. Pontifex M. G. Phelan M. M. Pidathala C. Kraemer B. C. Barclay J. W. (2018). α-Methyl-α-phenylsuccinimide Ameliorates Neurodegeneration in a *C. elegans* Model of TDP-43 Proteinopathy. Neurobiol. Dis. 118, 40–54. 10.1016/j.nbd.2018.06.013 29940336 PMC6097874

[B198] Wu J. Xu H. Tang W. Kopelman R. Philbert M. A. Xi C. (2009). Eradication of Bacteria in Suspension and Biofilms Using Methylene Blue-Loaded Dynamic Nanoplatforms. Antimicrob. Agents Chemother. 53, 3042–3048. 10.1128/AAC.01604-08 19414585 PMC2704698

[B199] Wu Y. Park K. C. Choi B. G. Park J. H. Yoon K. S. (2016). The Antibiofilm Effect of Ginkgo Biloba Extract against Salmonella and Listeria Isolates from Poultry. Foodborne Pathog. Dis. 13, 229–238. 10.1089/fpd.2015.2072 26954614

[B200] Wu Y. Wu Z. Butko P. Christen Y. Lambert M. P. Klein W. L. (2006). Amyloid-beta-induced Pathological Behaviors Are Suppressed by Ginkgo Biloba Extract EGb 761 and Ginkgolides in Transgenic *Caenorhabditis elegans* . J. Neurosci. 26, 13102–13113. 10.1523/JNEUROSCI.3448-06.2006 17167099 PMC6674971

[B201] Xiao L. Li H. Zhang J. Yang F. Huang A. Deng J. (2014). Salidroside Protects *Caenorhabditis elegans* Neurons from Polyglutamine-Mediated Toxicity by Reducing Oxidative Stress. Molecules 19, 7757–7769. 10.3390/molecules19067757 24918543 PMC6270757

[B202] Xin L. Yamujala R. Wang Y. Wang H. Wu W. H. Lawton M. A. (2013). Acetylcholineestarase-inhibiting Alkaloids from Lycoris Radiata Delay Paralysis of Amyloid Beta-Expressing Transgenic *C. elegans* CL4176. Plos One 8, e63874. 10.1371/journal.pone.0063874 23675513 PMC3652842

[B203] Yamada K. J. Heim C. E. Xi X. Attri K. S. Wang D. Zhang W. (2020). Monocyte Metabolic Reprogramming Promotes Pro-inflammatory Activity and *Staphylococcus aureus* Biofilm Clearance. Plos Pathog. 16, e1008354. 10.1371/journal.ppat.1008354 32142554 PMC7080272

[B204] Yang X. Zhang M. Dai Y. Sun Y. Aman Y. Xu Y. (2020). Spermidine Inhibits Neurodegeneration and Delays Aging via the PINK1-PDR1-dependent Mitophagy Pathway in *C. elegans* . Aging (Albany NY) 12, 16852–16866. 10.18632/aging.103578 32902411 PMC7521492

[B205] Yao C. Johnson W. M. Gao Y. Wang W. Zhang J. Deak M. (2013). Kinase Inhibitors Arrest Neurodegeneration in Cell and *C. elegans* Models of LRRK2 Toxicity. Hum. Mol. Genet. 22, 328–344. 10.1093/hmg/dds431 23065705 PMC3526163

[B206] You Z. Ran X. Dai Y. Ran Y. (2018). Clioquinol, an Alternative Antimicrobial Agent against Common Pathogenic Microbe. J. Mycol. Med. 28, 492–501. 10.1016/j.mycmed.2018.03.007 29650464

[B207] You Z. Zhang C. Ran Y. (2020). The Effects of Clioquinol in Morphogenesis, Cell Membrane and Ion Homeostasis in Candida Albicans. BMC Microbiol. 20, 165. 10.1186/s12866-020-01850-3 32546212 PMC7298956

[B208] Zaidi S. Singh S. L. Khan A. U. (2020). Exploring Antibiofilm Potential of Bacitracin against streptococcus Mutans. Microb. Pathog. 149, 104279. 10.1016/j.micpath.2020.104279 32512154

[B209] Zhang C. Liu Z. Bunker E. Ramirez A. Lee S. Peng Y. (2017). Sorafenib Targets the Mitochondrial Electron Transport Chain Complexes and ATP Synthase to Activate the PINK1-Parkin Pathway and Modulate Cellular Drug Response. J. Biol. Chem. 292, 15105–15120. 10.1074/jbc.M117.783175 28673964 PMC5592685

[B210] Zhang D. Anantharam V. Kanthasamy A. Kanthasamy A. G. (2007). Neuroprotective Effect of Protein Kinase C delta Inhibitor Rottlerin in Cell Culture and Animal Models of Parkinson's Disease. J. Pharmacol. Exp. Ther. 322, 913–922. 10.1124/jpet.107.124669 17565007

[B211] Zhang H. Su Y. Sun Z. Chen M. Han Y. Li Y. (2021). Ginsenoside Rg1 Alleviates Aβ Deposition by Inhibiting NADPH Oxidase 2 Activation in APP/PS1 Mice. J. Ginseng Res. 45, 665–675. 10.1016/j.jgr.2021.03.003 34764721 PMC8569324

[B212] Zhang S. Q. Obregon D. Ehrhart J. Deng J. Tian J. Hou H. (2013). Baicalein Reduces β-amyloid and Promotes Nonamyloidogenic Amyloid Precursor Protein Processing in an Alzheimer's Disease Transgenic Mouse Model. J. Neurosci. Res. 91, 1239–1246. 10.1002/jnr.23244 23686791 PMC3810722

[B213] Zhang W. He H. Song H. Zhao J. Li T. Wu L. (20162016). Neuroprotective Effects of Salidroside in the MPTP Mouse Model of Parkinson's Disease: Involvement of the PI3K/Akt/GSK3β Pathway. Parkinsons Dis. 2016, 9450137. 10.1155/2016/9450137 PMC5050371 27738547

[B214] Zhao W. X. Zhang J. H. Cao J. B. Wang W. Wang D. X. Zhang X. Y. (2017). Acetaminophen Attenuates Lipopolysaccharide-Induced Cognitive Impairment through Antioxidant Activity. J. Neuroinflammation 14, 17. 10.1186/s12974-016-0781-6 28109286 PMC5251335

[B215] Zheng C. Karimzadegan S. Chiang V. Chalfie M. (2013). Histone Methylation Restrains the Expression of Subtype-specific Genes during Terminal Neuronal Differentiation in *Caenorhabditis elegans* . Plos Genet. 9, e1004017. 10.1371/journal.pgen.1004017 24348272 PMC3861114

[B216] Zhou T. Zhu M. Liang Z. (2018). (-)-Epigallocatechin-3-gallate Modulates Peripheral Immunity in the MPTP-Induced Mouse Model of Parkinson's Disease. Mol. Med. Rep. 17, 4883–4888. 10.3892/mmr.2018.8470 29363729 PMC5865947

[B217] Zhou Y. Smith D. Leong B. J. Brännström K. Almqvist F. Chapman M. R. (2012). Promiscuous Cross-Seeding between Bacterial Amyloids Promotes Interspecies Biofilms. J. Biol. Chem. 287, 35092–35103. 10.1074/jbc.M112.383737 22891247 PMC3471717

