# Supplementary material for: Using Caenorhabditis elegans to Model Therapeutic Interventions of Neurodegenerative Diseases Targeting Microbe-Host Interactions
Source: Front Pharmacol. 2022 Apr 28;13:875349. doi: 10.3389/fphar.2022.875349 (PMC9096141; doi:10.3389/fphar.2022.875349)
Supplement: Supplementary file 1 [file Table1.docx]

**Table S1. Compounds with neuroprotective effects in neurodegenerative disease models showed inhibitory effects on microorganisms.** Asterisks (*) mark the compounds that could inhibit the bacterial biofilm formation. ND means “not determined”.

| **Compounds** | **Worm models** | | **Mouse model** | | **Known effects on the microorganism** |
| --- | --- | --- | --- | --- | --- |
|  | **Diseases** | **Neuroprotective effects** | **Diseases** | **Neuroprotective effects** |  |
| Ginkgo biloba extract* | AD | Reduce Aβ deposit, Aβ-induced paralysis, and chemotaxis (Wu et al., 2006) | AD | Reduce Aβ toxicity and improves cognitive functions (Tchantchou et al., 2007) | Inhibit biofilm formation (Wu et al., 2016) |
| Caffeine* | AD | Reduce Aβ-induced paralysis and increase antioxidant capacity (Dostal et al., 2010) | AD | Reduce dementia (Arendash et al., 2006; Eskelinen and Kivipelto, 2010) | Inhibit bacteria growth at high dose; inhibit biofilm formation and cause biofilm dispersal (Chakraborty et al., 2020; Sandlie et al., 1980) |
| Clioquinol | AD | Reduce Aβ accumulation and enhance memory formation (Matlack et al., 2014) | AD | Reduce Aβ plaque burden and attenuate astrogliosis (Grossi et al., 2009) | Inhibit fungal biofilm formation (You et al., 2018; You et al., 2020) |
| Curcumin* | AD | Reduce Aβ accumulation and increase lifespan (Alavez et al., 2011; Miyasaka et al., 2016) | AD | Reduce Aβ accumulation, oxidative damage, and inflammation (Lim et al., 2001); inhibit neuronal death and iNOS expression in AD models (Begum et al., 2008) | Inhibit biofilm formation (Kali et al., 2016) and induce biofilm dispersal (Ding et al., 2017) |
| Ferulic acid* | AD | Reduce Aβ accumulation and enhance autophagy (Wang et al., 2020) | AD | Reduce vasoconstriction of hippocampal capillaries and ameliorate Aβ plaque deposition and spatial memory deficit (Wang et al., 2021b) | Inhibit bacteria growth and inhibit biofilm formation (Borges et al., 2012; Takahashi et al., 2013); induce biofilm dispersal (Dasagrandhi et al., 2018) |
| Fluoxetine* | AD | Reduce Aβ-induced paralysis; enhance thermal resistance and increase lifespan (Keowkase et al., 2010b) | AD | Reduce APP cleavage and Aβ generation and neuronal apoptosis (Huang et al., 2018) | Modulate bacterial gut colonization and inhibit biofilm formation (Fung et al., 2019; Pelling et al., 2019) |
| Galanthamine | AD | Reduce Aβ-induced paralysis; increase lifespan (Xin et al., 2013) | AD | Slow down Aβ plaque formation and behavioral decline (Bhattacharya et al., 2014) | ND |
| Glycitein | AD | Reduce Aβ deposits and ROS production (Gutierrez-Zepeda et al., 2005) |  | ND | Among the antibacterial components of Doenjang extracts (Lalouckova et al., 2021) |
| JAY2-22-33 | AD | Reduce Aβ-induced paralysis (Keowkase et al., 2010a) |  | ND | ND |
| JWB1-84-1 | AD | Reduce Aβ-induced paralysis (Keowkase *et al.*, 2010a) | AD | Improve memory-related task accuracy (Sood et al., 2007) | ND |
| Quercetin* | AD | Decrease Aβ production (Regitz et al., 2014) | PD | Protect against DA neurodegeneration and mitochondrial dysfunction (Ay et al., 2017) | Inhibit biofilm formation (Memariani et al., 2019) |
| Rifampicin* | AD | Reduce Aβ-induced paralysis (Lublin et al., 2011) | AD | Inhibit Aβ oligomerization, tau hyperphosphorylation, and synapse loss and Enhance memory formation  (Umeda et al., 2018) | Antibiotic; inhibit biofilm formation (Verma et al., 2021) |
| Tannic acid* | AD | Increase lifespan and reduce Aβ-amyloid fibril (Lublin *et al.*, 2011) | AD | Inhibit β-secretase and prevents Aβ-induced cognitive impairment (Takashi Mori, 2012) | Inhibit bacterial growth and biofilm formation (Dong et al., 2018); induce biofilm dispersal (Siddiquia, 2019) |
| Tetracycline* | AD | Reduce Aβ oligomer deposition and inhibit Aβ-induced paralysis and oxidative stress (Diomede et al., 2010) | AD | Reduce the level of Aβ oligomers and improve memory (Balducci et al., 2018) | Antibiotic; inhibit biofilm formation (Stone et al., 2002) |
| Thioflavin T* | AD | Increase lifespan and locomotion and reduce tau toxicity (Gamir-Morralla et al., 2019) | AD | Reduce amyloid plaques and inhibit microglial activation (Sarkar et al., 2015) | Inhibit biofilm formation (Bondia et al., 2021) |
| Acetylcorynoline | AD, PD | Reduce α-synuclein aggregation, DA neuron degeneration and enhance proteasome activity (Fu et al., 2014) |  | ND | ND |
| Bacitracin* | AD, PD | Reduce Aβ-induced motor deficits and aging (Lublin *et al.*, 2011); inhibit MPP(+)-induced dopaminergic (DA) neuron loss (Lehtonen et al., 2016) | PD | Attenuate motor deficits, decrease DA neuron loss, and reduce inflammation in 6-OHDA-treated rats (Koutzoumis et al., 2020) | Antibiotic; inhibit biofilm formation (Zaidi et al., 2020) |
| EGCG* | AD, PD | Reduce Aβ oligomer deposits and formation of lipofuscin (Abbas and Wink, 2010); reduce α-synuclein-induced locomotion defects (Wang et al., 2021a) | ALS, PD, AD | Prolong symptom onset and lifespan in ALS model (Koh et al., 2006); decrease inflammation in MPTP-induced PD model (Zhou et al., 2018); restore mitochondrial function and ATP levels in AD model (Dragicevic et al., 2011) | Inhibit biofilm formation and induce biofilm dispersal (Serra et al., 2016) |
| Valproic acid | AD, PD | Reduce Aβ deposition and memory deficits (Evason et al., 2008); inhibit α-synuclein-induced DA neuron loss(Kautu et al., 2013) | PD | Inhibit MPTP-induced DA neuron loss (Kidd and Schneider, 2011) | Inhibit fungal growth and fungal biofilm formation (Singh et al., 2021) |
| Acetaminophen* | PD | Prolong lifespan (Lublin *et al.*, 2011), inhibit α-synuclein-induced DA neuron loss and locomotion defects (Chen et al., 2021; Locke et al., 2008) |  | Reduce LPS-induced cognitive impairment and hippocampal damage (Zhao et al., 2017) | Inhibit biofilm formation (Abidi et al., 2019) |
| Losartan | PD | Inhibit α-synuclein-induced locomotion defects (Chen *et al.*, 2021) | PD | Reduce α-synuclein oligomers in PD rat neurons (Chen *et al.*, 2021) | ND |
| Rifabutin* | PD | Inhibit α-synuclein-induced locomotion defects (Chen *et al.*, 2021) | PD | Reduce α-synuclein oligomers in PD rat neurons (Chen *et al.*, 2021) | Inhibit bacterial biofilm and infection (Doub et al., 2020) |
| Spermidine | AD, PD | Reduce α-synuclein accumulation, improve locomotion, and activate mitophagy in PD and reduce memory loss in AD (Buttner et al., 2014; Yang et al., 2020) | FTLD | Activate autophagy, reduce TDP-43-induced neuron loss, and enhance performance in rotarod tests (Wang et al., 2012) | Promote biofilm formation (Hobley et al., 2017; Thongbhubate et al., 2021) |
| Metformin* | AD, PD, HD | Rescue 6-OHDA-induced DA neuron loss (Saewanee et al., 2021); inhibit Aβ-induced paralysis and improve neurotransmitter function (Ahmad and Ebert, 2017); reduce polyQ aggregation (Sanchis et al., 2019) | AD, HD, PD | Reduce behavioral defects in HD mice (Sanchis *et al.*, 2019); improve learning and memory in AD mice (Farr et al., 2019); rescue MPTP-induced neurodegeneration and increase BDNF levels (Patil et al., 2014) | Inhibit bacterial biofilm and quorum sensing (Abbas et al., 2017) |
| Icariin and its derivative icariside II* | AD, HD | Increase tolerance to thermo and oxidative stress; delay the onset of polyQ or Aβ -induced paralysis (Cai et al., 2011) | AD | Improve cognitive function; reduce neuronal apoptosis and suppress ER stress in AD (Li et al., 2019) | Inhibit biofilm formation (Coenye et al., 2012) |
| PBT2 | AD, HD | Inhibit polyQ- and Aβ-induced paralysis (Cherny et al., 2012; McColl et al., 2012) | AD, HD | Reduce tau accumulation and enhance cognition (Sedjahtera et al., 2018); reduces brain weight loss and enhance motor performance in HD mice (Cherny *et al.*, 2012) | Inhibit polymyxin-resistance of Gram-negative pathogens (De Oliveira et al., 2020) |
| Apomorphine | PD | Rescue MPP(+)-induced locomotion defects (Mocko et al., 2010) | AD | Reduce Aβ accumulation and tau hyperphosphorylation and enhance memory formation (Himeno et al., 2011) | ND |
| Baicalin* | PD | Alleviate 6-OHDA-induced oxidative stress; improve locomotion and survival (Ma et al., 2021) | AD | Reduce Aβ production and improve cognitive function (Zhang et al., 2013) | Antimicrobial activity; inhibit biofilm formation (Luo et al., 2017) |
| Bromocriptine | PD | Rescue MPP(+)-induced locomotion defects (Mocko *et al.*, 2010) | PD | Reduce 6-OHDA-induced DA neuron loss (Ogawa et al., 1994) | ND |
| Betulin* | PD | Reduce α-synuclein accumulation and inhibit 6-OHDA-induced DA neurodegeneration (Tsai et al., 2017) | AD | Mitigate scopolamine-induced memory deficits (Cho et al., 2016) | Inhibit biofilm formation (Viszwapriya et al., 2016) |
| Indoline and its derivative GW5074 | PD | Reduce LRRK2(G2019S)-induced DA neurodegeneration (Liu et al., 2011) | HD | Inhibit 3-Nitropropionic acid-induced neurodegeneration and behavioral deficits (Chin et al., 2004) | Inhibit gram-positive bacteria growth (Clement Opoku-Temeng, 2017) |
| Ginsenoside* | PD | Attenuate 6-OHDA-induced DA neurodegeneration (Chalorak et al., 2021) | AD | Alleviate cognitive dysfunction and neuronal damage and decrease Aβ and phosphorylated tau levels (Zhang et al., 2021) | Antibiofilm activity; induce biofilm dispersion (Cao et al., 2019) |
| Lisuride | PD | Inhibit MPP(+)-induced neurodegeneration (Braungart et al., 2004) | PD | Increase wakefulness and paradoxical sleep latency in MPTP-treated mice (Laloux et al., 2008) | ND |
| LRRK2-IN1 | PD | Reduce LRRK2 mutants-induced DA neurodegeneration (Yao et al., 2013) | PD | Inhibit LRRK2 mutants-induced DA neurodegeneration but incapable of permeating the blood-brain barrier (Chen et al., 2018) | ND |
| P7C3 | PD | Block MPP(+)-mediated DA neuron death (De Jesus-Cortes et al., 2012) | PD | Inhibits microglial activation and DA neuronal loss (Gu et al., 2018) | ND |
| Rottlerin* | PD | Ameliorate MPP(+)-induced neurodegeneration (Braungart *et al.*, 2004) | PD | Inhibit MPTP-induced locomotor deficits and striatal depletion of dopamine (Zhang et al., 2007) | Inhibit bacterial quorum sensing and biofilm formation (Suresh et al., 2021) |
| Sorafenib and its derivative* | PD | Inhibit LRRK2(G2019S)-induced DA neurodegeneration (Liu *et al.*, 2011) | PD | Activate PINK1-Parkin pathway (Zhang et al., 2017) | Inhibit biofilm formation (Cui et al., 2019) |
| Tauroursodeoxycholic acid | PD | Rescue the increased vulnerability to rotenone in DJ-1 knockout mutants (Ved et al., 2005) | PD | Reduce α-synuclein aggregation, DA neurodegeneration and prevent glial activation, oxidation, and autophagy (Cuevas et al., 2020) | ND |
| TTT-3002 | PD | Reduce LRRK2 mutants-induced DA neurodegeneration (Yao *et al.*, 2013) |  | ND | ND |
| Celecoxib* | HD | Delay the onset of polyQ-mediated protein aggregation and proteotoxicity(Ching et al., 2011) | PD | Reduced LPS-induced sensorimotor behavioral defects and DA neuron dysfunction (Kaizaki et al., 2013) | Inhibit biofilm formation (Tzeng et al., 2020) |
| Lithium | HD | Ameliorate polyQ-mediated neuron loss (Voisine et al., 2007) | HD | Alleviated spontaneous locomotor deficits and depressive-like behaviors in HD mice (Chiu et al., 2011) | Absorbed by biofilm polymer (Andi Kurniawan, 2013) |
| Mithramycin | HD | Ameliorate polyQ-mediated neuron loss (Voisine *et al.*, 2007) | HD | Reduce neuropathology and increase lifespan in HD mice (Ferrante et al., 2004) | Produced by bacteria (Pham et al., 2019) |
| ML346* | HD | Activates transcription of the Hsp70 promoter and suppresses aggregation of polyQ (Calamini et al., 2010) |  | ND | Inhibit biofilm formation (Guan X, 2022) |
| Oligomycin* | HD | Dose-dependent rescue of polyQ induced neuronal death (Varma et al., 2007) |  | ND | Antibiotic; clear established biofilm (Yamada et al., 2020) |
| Rotenone | HD | Dose-dependent rescue of polyQ induced neuronal death (Varma *et al.*, 2007) |  | Induce DA neurodegeneration at 30 mg/mL (Inden et al., 2011) | ND |
| Salidroside* | HD | Ameliorate polyQ-mediated neuron loss, increase antioxidant capacity, and reduce paraquat-induced mortality (Xiao et al., 2014) | PD | Reduce MPTP-induced motor defects and rescue the loss of tyrosine hydroxylase (TH)-positive neurons (Zhang et al., 2016) | Inhibit biofilm formation (Coenye *et al.*, 2012) |
| Trichostatin A and other HDAC inhibitors | HD, PD | Ameliorate polyQ-mediated neuron loss (Bates et al., 2006; Voisine *et al.*, 2007) | PD | Increase the expression of TH and BDNF and protect the nigrostriatal DA neurons in MPTP-treated mice (Suo et al., 2015) | Inhibit fungal biofilm formation (Cécile Garnaud, 2016) |
| Azaperone or isoniazid | FTDP | Reduce insoluble tau and inhibit tau-induced locomotion defects and neuronal loss (McCormick et al., 2013) | FTDP | Reduce the level of insoluble tau (Crowe et al., 2020) | ND |
| Perphenazine | FTDP | Reduce insoluble tau and inhibit tau-induced locomotion defects and neuronal loss (McCormick *et al.*, 2013) |  | ND | ND |
| Trazodone | FTDP | Reduce insoluble tau and inhibit tau-induced locomotion defects and neuronal loss (McCormick *et al.*, 2013) | FTDP | Reduced the burden of hyperphosphorylated tau (Halliday et al., 2017) | ND |
| Zotepine | FTDP | Reduce insoluble tau and inhibit tau-induced locomotion defects and neuronal loss (McCormick *et al.*, 2013) |  | ND | Inhibit fungal biofilm formation (Siles et al., 2013) |
| Guanabenz | ALS | Reduce TDP-43-induced paralysis, neurodegeneration, oxidative stress, and ER stress response (Vaccaro et al., 2013) | ALS | Protect fibroblasts expressing SOD1(G93A) mutants during ER stress (Vieira et al., 2015) | ND |
| Propyl gallate* | ALS | Reduce TDP-43-induced paralysis and axonal degeneration and increase lifespan (Tauffenberger et al., 2013) | AD | Inhibit Aβ aggregation (Chan et al., 2016) | Inhibit biofilm formation (Kosuru et al., 2021) |
| Salubrinal | ALS | Reduce TDP-43-induced paralysis, neurodegeneration, oxidative stress, and ER stress response (Vaccaro *et al.*, 2013) | ALS | Inhibit eIF2a dephosphorylation and reduce ER stress; ameliorate SOD1 mutants-induced disease symptoms and extend survival (Saxena et al., 2009) | ND |
| Trolox | ALS | Reduce TDP-43-induced paralysis and axonal degeneration and increase lifespan (Tauffenberger *et al.*, 2013) | ALS | Reduce cytosolic oxidative stress in SOD1 neurotoxicity (Rojas et al., 2015) | ND |
| α-methyl-α-phenylsuccinimide | ALS | Ameliorates TDP-43-induced locomotion defects and neurodegeneration (Wong et al., 2018) |  | ND | ND |
| Methylene blue* | ALS, FTDP | Reduce detergent insoluble phospho-tau and protect against TDP-43 neurotoxicity (Fatouros et al., 2012; Vaccaro et al., 2012a; Vaccaro et al., 2012b) | FTDP | Reduce detergent insoluble phospho-tau (Hosokawa et al., 2012) | Visualize biofilm; inhibit biofilm formation; induce biofilm dispersal (Shaw et al., 2020; Wu et al., 2009) |
| PHA767491 | ALS | Reduce TDP-43 phosphorylation and prevent TDP-43-induced neurodegeneration (Liachko et al., 2013) | ALS | Ameliorate the gait ataxia and inflammatory response in TDP-43 transgenic mice (Chung et al., 2020) | ND |
| LDN-0130436 | ALS | Inhibit TDP-43 aggregation in a dose-dependent manner (Boyd et al., 2014) |  | ND | ND |

**References:**

Abbas, H.A., Elsherbini, A.M., and Shaldam, M.A. (2017). Repurposing metformin as a quorum sensing inhibitor in Pseudomonas aeruginosa. Afr Health Sci *17*, 808-819. 10.4314/ahs.v17i3.24.

Abbas, S., and Wink, M. (2010). Epigallocatechin gallate inhibits beta amyloid oligomerization in Caenorhabditis elegans and affects the daf-2/insulin-like signaling pathway. Phytomedicine *17*, 902-909. 10.1016/j.phymed.2010.03.008.

Abidi, S.H., Ahmed, K., and Kazmi, S.U. (2019). The antibiofilm activity of Acetylsalicylic acid, Mefenamic acid, Acetaminophen against biofilms formed by P. aeruginosa and S. epidermidis. J Pak Med Assoc *69*, 1493-1495.

Ahmad, W., and Ebert, P.R. (2017). Metformin Attenuates Abeta Pathology Mediated Through Levamisole Sensitive Nicotinic Acetylcholine Receptors in a C. elegans Model of Alzheimer's Disease. Mol Neurobiol *54*, 5427-5439. 10.1007/s12035-016-0085-y.

Alavez, S., Vantipalli, M.C., Zucker, D.J., Klang, I.M., and Lithgow, G.J. (2011). Amyloid-binding compounds maintain protein homeostasis during ageing and extend lifespan. Nature *472*, 226-229. 10.1038/nature09873.

Andi Kurniawan, T.Y. (2013). Biofilm Polymer for Biosorption of Pollutant. Procedia Environmental Sciences *17*, 179-187. 10.1016/j.proenv.2013.02.027.

Arendash, G.W., Schleif, W., Rezai-Zadeh, K., Jackson, E.K., Zacharia, L.C., Cracchiolo, J.R., Shippy, D., and Tan, J. (2006). Caffeine protects Alzheimer's mice against cognitive impairment and reduces brain beta-amyloid production. Neuroscience *142*, 941-952. 10.1016/j.neuroscience.2006.07.021.

Ay, M., Luo, J., Langley, M., Jin, H., Anantharam, V., Kanthasamy, A., and Kanthasamy, A.G. (2017). Molecular mechanisms underlying protective effects of quercetin against mitochondrial dysfunction and progressive dopaminergic neurodegeneration in cell culture and MitoPark transgenic mouse models of Parkinson's Disease. J Neurochem *141*, 766-782. 10.1111/jnc.14033.

Balducci, C., Santamaria, G., La Vitola, P., Brandi, E., Grandi, F., Viscomi, A.R., Beeg, M., Gobbi, M., Salmona, M., Ottonello, S., and Forloni, G. (2018). Doxycycline counteracts neuroinflammation restoring memory in Alzheimer's disease mouse models. Neurobiol Aging *70*, 128-139. 10.1016/j.neurobiolaging.2018.06.002.

Bates, E.A., Victor, M., Jones, A.K., Shi, Y., and Hart, A.C. (2006). Differential contributions of Caenorhabditis elegans histone deacetylases to huntingtin polyglutamine toxicity. J Neurosci *26*, 2830-2838. 10.1523/JNEUROSCI.3344-05.2006.

Begum, A.N., Jones, M.R., Lim, G.P., Morihara, T., Kim, P., Heath, D.D., Rock, C.L., Pruitt, M.A., Yang, F., Hudspeth, B., et al. (2008). Curcumin structure-function, bioavailability, and efficacy in models of neuroinflammation and Alzheimer's disease. J Pharmacol Exp Ther *326*, 196-208. 10.1124/jpet.108.137455.

Bhattacharya, S., Haertel, C., Maelicke, A., and Montag, D. (2014). Galantamine slows down plaque formation and behavioral decline in the 5XFAD mouse model of Alzheimer's disease. Plos One *9*, e89454. 10.1371/journal.pone.0089454.

Bondia, P., Flors, C., and Torra, J. (2021). Boosting the inactivation of bacterial biofilms by photodynamic targeting of matrix structures with Thioflavin T. Chem Commun *57*, 8648-8651. 10.1039/d1cc03155d.

Borges, A., Saavedra, M.J., and Simoes, M. (2012). The activity of ferulic and gallic acids in biofilm prevention and control of pathogenic bacteria. Biofouling *28*, 755-767. 10.1080/08927014.2012.706751.

Boyd, J.D., Lee, P., Feiler, M.S., Zauur, N., Liu, M., Concannon, J., Ebata, A., Wolozin, B., and Glicksman, M.A. (2014). A high-content screen identifies novel compounds that inhibit stress-induced TDP-43 cellular aggregation and associated cytotoxicity. J Biomol Screen *19*, 44-56. 10.1177/1087057113501553.

Braungart, E., Gerlach, M., Riederer, P., Baumeister, R., and Hoener, M.C. (2004). Caenorhabditis elegans MPP+ model of Parkinson's disease for high-throughput drug screenings. Neurodegener Dis *1*, 175-183. 10.1159/000080983.

Buttner, S., Broeskamp, F., Sommer, C., Markaki, M., Habernig, L., Alavian-Ghavanini, A., Carmona-Gutierrez, D., Eisenberg, T., Michael, E., Kroemer, G., et al. (2014). Spermidine protects against alpha-synuclein neurotoxicity. Cell Cycle *13*, 3903-3908. 10.4161/15384101.2014.973309.

Cai, W.J., Huang, J.H., Zhang, S.Q., Wu, B., Kapahi, P., Zhang, X.M., and Shen, Z.Y. (2011). Icariin and its derivative icariside II extend healthspan via insulin/IGF-1 pathway in C. elegans. Plos One *6*, e28835. 10.1371/journal.pone.0028835.

Calamini, B., Silva, M.C., Madoux, F., Hutt, D.M., Khanna, S., Chalfant, M.A., Allais, C., Ouizem, S., Saldanha, S.A., Ferguson, J., et al. (2010). ML346: A Novel Modulator of Proteostasis for Protein Conformational Diseases. In Probe Reports from the NIH Molecular Libraries Program.

Cao, X., Ye, Q., Fan, M., and Liu, C. (2019). Antimicrobial effects of the ginsenoside Rh2 on monospecies and multispecies cariogenic biofilms. J Appl Microbiol *126*, 740-751. 10.1111/jam.14178.

Cécile Garnaud, M.C., Danièle Maubon, Muriel Cornet, and Jérôme Govin (2016). Histone Deacetylases and Their Inhibition in Candida Species. Front Microbiol. *7*. 10.3389/fmicb.2016.01238.

Chakraborty, P., Dastidar, D.G., Paul, P., Dutta, S., Basu, D., Sharma, S.R., Basu, S., Sarker, R.K., Sen, A., Sarkar, A., and Tribedi, P. (2020). Inhibition of biofilm formation of Pseudomonas aeruginosa by caffeine: a potential approach for sustainable management of biofilm. Arch Microbiol *202*, 623-635. 10.1007/s00203-019-01775-0.

Chalorak, P., Sanguanphun, T., Limboonreung, T., and Meemon, K. (2021). Neurorescue Effects of Frondoside A and Ginsenoside Rg3 in C. elegans Model of Parkinson's Disease. Molecules *26*. 10.3390/molecules26164843.

Chan, S., Kantham, S., Rao, V.M., Palanivelu, M.K., Pham, H.L., Shaw, P.N., McGeary, R.P., and Ross, B.P. (2016). Metal chelation, radical scavenging and inhibition of Abeta(4)(2) fibrillation by food constituents in relation to Alzheimer's disease. Food Chem *199*, 185-194. 10.1016/j.foodchem.2015.11.118.

Chen, J., Chen, Y., and Pu, J. (2018). Leucine-Rich Repeat Kinase 2 in Parkinson's Disease: Updated from Pathogenesis to Potential Therapeutic Target. Eur Neurol *79*, 256-265. 10.1159/000488938.

Chen, K.S., Menezes, K., Rodgers, J.B., O'Hara, D.M., Tran, N., Fujisawa, K., Ishikura, S., Khodaei, S., Chau, H., Cranston, A., et al. (2021). Small molecule inhibitors of alpha-synuclein oligomers identified by targeting early dopamine-mediated motor impairment in C. elegans. Mol Neurodegener *16*, 77. 10.1186/s13024-021-00497-6.

Cherny, R.A., Ayton, S., Finkelstein, D.I., Bush, A.I., McColl, G., and Massa, S.M. (2012). PBT2 Reduces Toxicity in a C. elegans Model of polyQ Aggregation and Extends Lifespan, Reduces Striatal Atrophy and Improves Motor Performance in the R6/2 Mouse Model of Huntington's Disease. J Huntingtons Dis *1*, 211-219. 10.3233/JHD-120029.

Chin, P.C., Liu, L., Morrison, B.E., Siddiq, A., Ratan, R.R., Bottiglieri, T., and D'Mello, S.R. (2004). The c-Raf inhibitor GW5074 provides neuroprotection in vitro and in an animal model of neurodegeneration through a MEK-ERK and Akt-independent mechanism. J Neurochem *90*, 595-608. 10.1111/j.1471-4159.2004.02530.x.

Ching, T.T., Chiang, W.C., Chen, C.S., and Hsu, A.L. (2011). Celecoxib extends C. elegans lifespan via inhibition of insulin-like signaling but not cyclooxygenase-2 activity. Aging Cell *10*, 506-519. 10.1111/j.1474-9726.2011.00688.x.

Chiu, C.T., Liu, G., Leeds, P., and Chuang, D.M. (2011). Combined treatment with the mood stabilizers lithium and valproate produces multiple beneficial effects in transgenic mouse models of Huntington's disease. Neuropsychopharmacology *36*, 2406-2421. 10.1038/npp.2011.128.

Cho, N., Kim, H.W., Lee, H.K., Jeon, B.J., and Sung, S.H. (2016). Ameliorative effect of betulin from Betula platyphylla bark on scopolamine-induced amnesic mice. Biosci Biotechnol Biochem *80*, 166-171. 10.1080/09168451.2015.1072460.

Chung, Y.H., Lin, C.W., Huang, H.Y., Chen, S.L., Huang, H.J., Sun, Y.C., Lee, G.C., Lee-Chen, G.J., Chang, Y.C., and Hsieh-Li, H.M. (2020). Targeting Inflammation, PHA-767491 Shows a Broad Spectrum in Protein Aggregation Diseases. J Mol Neurosci *70*, 1140-1152. 10.1007/s12031-020-01521-y.

Clement Opoku-Temeng, N.D., Jacob Miller and Herman O. Sintim (2017). Hydroxybenzylidene-indolinones, c-di-AMP synthase inhibitors, have antibacterial and anti-biofilm activities and also re-sensitize resistant bacteria to methicillin and vancomycin. The Royal Society of Chemistry *7*. 10.1039/c6ra28443d.

Coenye, T., Brackman, G., Rigole, P., De Witte, E., Honraet, K., Rossel, B., and Nelis, H.J. (2012). Eradication of Propionibacterium acnes biofilms by plant extracts and putative identification of icariin, resveratrol and salidroside as active compounds. Phytomedicine *19*, 409-412. 10.1016/j.phymed.2011.10.005.

Crowe, A., Henderson, M.J., Anderson, J., Titus, S.A., Zakharov, A., Simeonov, A., Buist, A., Delay, C., Moechars, D., Trojanowski, J.Q., et al. (2020). Compound screening in cell-based models of tau inclusion formation: Comparison of primary neuron and HEK293 cell assays. J Biol Chem *295*, 4001-4013. 10.1074/jbc.RA119.010532.

Cuevas, E., Burks, S., Raymick, J., Robinson, B., Gomez-Crisostomo, N.P., Escudero-Lourdes, C., Lopez, A.G.G., Chigurupati, S., Hanig, J., Ferguson, S.A., and Sarkar, S. (2020). Tauroursodeoxycholic acid (TUDCA) is neuroprotective in a chronic mouse model of Parkinson's disease. Nutr Neurosci, 1-18. 10.1080/1028415X.2020.1859729.

Cui, W.Q., Qu, Q.W., Wang, J.P., Bai, J.W., Bello-Onaghise, G., Li, Y.A., Zhou, Y.H., Chen, X.R., Liu, X., Zheng, S.D., et al. (2019). Discovery of Potential Anti-infective Therapy Targeting Glutamine Synthetase in Staphylococcus xylosus. Front Chem *7*, 381. 10.3389/fchem.2019.00381.

Dasagrandhi, C., Park, S., Jung, W.K., and Kim, Y.M. (2018). Antibacterial and Biofilm Modulating Potential of Ferulic Acid-Grafted Chitosan against Human Pathogenic Bacteria. Int J Mol Sci *19*. 10.3390/ijms19082157.

De Jesus-Cortes, H., Xu, P., Drawbridge, J., Estill, S.J., Huntington, P., Tran, S., Britt, J., Tesla, R., Morlock, L., Naidoo, J., et al. (2012). Neuroprotective efficacy of aminopropyl carbazoles in a mouse model of Parkinson disease. Proc Natl Acad Sci U S A *109*, 17010-17015. 10.1073/pnas.1213956109.

De Oliveira, D.M.P., Bohlmann, L., Conroy, T., Jen, F.E., Everest-Dass, A., Hansford, K.A., Bolisetti, R., El-Deeb, I.M., Forde, B.M., Phan, M.D., et al. (2020). Repurposing a neurodegenerative disease drug to treat Gram-negative antibiotic-resistant bacterial sepsis. Sci Transl Med *12*. 10.1126/scitranslmed.abb3791.

Ding, T., Li, T., Wang, Z., and Li, J. (2017). Curcumin liposomes interfere with quorum sensing system of Aeromonas sobria and in silico analysis. Sci Rep *7*, 8612. 10.1038/s41598-017-08986-9.

Diomede, L., Cassata, G., Fiordaliso, F., Salio, M., Ami, D., Natalello, A., Doglia, S.M., De Luigi, A., and Salmona, M. (2010). Tetracycline and its analogues protect Caenorhabditis elegans from beta amyloid-induced toxicity by targeting oligomers. Neurobiol Dis *40*, 424-431. 10.1016/j.nbd.2010.07.002.

Dong, G., Liu, H., Yu, X., Zhang, X., Lu, H., Zhou, T., and Cao, J. (2018). Antimicrobial and anti-biofilm activity of tannic acid against Staphylococcus aureus. Nat Prod Res *32*, 2225-2228. 10.1080/14786419.2017.1366485.

Dostal, V., Roberts, C.M., and Link, C.D. (2010). Genetic mechanisms of coffee extract protection in a Caenorhabditis elegans model of beta-amyloid peptide toxicity. Genetics *186*, 857-866. 10.1534/genetics.110.120436.

Doub, J.B., Heil, E.L., Ntem-Mensah, A., Neeley, R., and Ching, P.R. (2020). Rifabutin Use in Staphylococcus Biofilm Infections: A Case Series. Antibiotics (Basel) *9*. 10.3390/antibiotics9060326.

Dragicevic, N., Smith, A., Lin, X., Yuan, F., Copes, N., Delic, V., Tan, J., Cao, C., Shytle, R.D., and Bradshaw, P.C. (2011). Green tea epigallocatechin-3-gallate (EGCG) and other flavonoids reduce Alzheimer's amyloid-induced mitochondrial dysfunction. J Alzheimers Dis *26*, 507-521. 10.3233/JAD-2011-101629.

Eskelinen, M.H., and Kivipelto, M. (2010). Caffeine as a Protective Factor in Dementia and Alzheimer's Disease. J Alzheimers Dis *20*, S167-S174. 10.3233/Jad-2010-1404.

Evason, K., Collins, J.J., Huang, C., Hughes, S., and Kornfeld, K. (2008). Valproic acid extends Caenorhabditis elegans lifespan. Aging Cell *7*, 305-317. 10.1111/j.1474-9726.2008.00375.x.

Farr, S.A., Roesler, E., Niehoff, M.L., Roby, D.A., McKee, A., and Morley, J.E. (2019). Metformin Improves Learning and Memory in the SAMP8 Mouse Model of Alzheimer's Disease. J Alzheimers Dis *68*, 1699-1710. 10.3233/JAD-181240.

Fatouros, C., Pir, G.J., Biernat, J., Koushika, S.P., Mandelkow, E., Mandelkow, E.M., Schmidt, E., and Baumeister, R. (2012). Inhibition of tau aggregation in a novel Caenorhabditis elegans model of tauopathy mitigates proteotoxicity. Hum Mol Genet *21*, 3587-3603. 10.1093/hmg/dds190.

Ferrante, R.J., Ryu, H., Kubilus, J.K., D'Mello, S., Sugars, K.L., Lee, J., Lu, P., Smith, K., Browne, S., Beal, M.F., et al. (2004). Chemotherapy for the brain: the antitumor antibiotic mithramycin prolongs survival in a mouse model of Huntington's disease. J Neurosci *24*, 10335-10342. 10.1523/JNEUROSCI.2599-04.2004.

Fu, R.H., Wang, Y.C., Chen, C.S., Tsai, R.T., Liu, S.P., Chang, W.L., Lin, H.L., Lu, C.H., Wei, J.R., Wang, Z.W., et al. (2014). Acetylcorynoline attenuates dopaminergic neuron degeneration and alpha-synuclein aggregation in animal models of Parkinson's disease. Neuropharmacology *82*, 108-120. 10.1016/j.neuropharm.2013.08.007.

Fung, T.C., Vuong, H.E., Luna, C.D.G., Pronovost, G.N., Aleksandrova, A.A., Riley, N.G., Vavilina, A., McGinn, J., Rendon, T., Forrest, L.R., and Hsiao, E.Y. (2019). Intestinal serotonin and fluoxetine exposure modulate bacterial colonization in the gut. Nat Microbiol *4*, 2064-2073. 10.1038/s41564-019-0540-4.

Gamir-Morralla, A., Sacristan, S., Medina, M., and Iglesias, T. (2019). Effects of Thioflavin T and GSK-3 Inhibition on Lifespan and Motility in a Caenorhabditis elegans Model of Tauopathy. J Alzheimers Dis Rep *3*, 47-57. 10.3233/ADR-180087.

Grossi, C., Francese, S., Casini, A., Rosi, M.C., Luccarini, I., Fiorentini, A., Gabbiani, C., Messori, L., Moneti, G., and Casamenti, F. (2009). Clioquinol decreases amyloid-beta burden and reduces working memory impairment in a transgenic mouse model of Alzheimer's disease. J Alzheimers Dis *17*, 423-440. 10.3233/JAD-2009-1063.

Gu, C., Hu, Q., Wu, J., Mu, C., Ren, H., Liu, C.F., and Wang, G. (2018). P7C3 Inhibits LPS-Induced Microglial Activation to Protect Dopaminergic Neurons Against Inflammatory Factor-Induced Cell Death in vitro and in vivo. Front Cell Neurosci *12*, 400. 10.3389/fncel.2018.00400.

Guan X, Z.T., . Yang T,. Dong Z,. Yang S, Lan L., Gane J., Yang C. (2022). Covalent sortase A inhibitor ML346 prevents Staphylococcus aureus infection of Galleria mellonella. RSC Medicinal Chemistry *13*, 138-149. 10.1039/d1md00316j.

Gutierrez-Zepeda, A., Santell, R., Wu, Z., Brown, M., Wu, Y., Khan, I., Link, C.D., Zhao, B., and Luo, Y. (2005). Soy isoflavone glycitein protects against beta amyloid-induced toxicity and oxidative stress in transgenic Caenorhabditis elegans. BMC Neurosci *6*, 54. 10.1186/1471-2202-6-54.

Halliday, M., Radford, H., Zents, K.A.M., Molloy, C., Moreno, J.A., Verity, N.C., Smith, E., Ortori, C.A., Barrett, D.A., Bushell, M., and Mallucci, G.R. (2017). Repurposed drugs targeting eIF2&alpha;-P-mediated translational repression prevent neurodegeneration in mice. Brain *140*, 1768-1783. 10.1093/brain/awx074.

Himeno, E., Ohyagi, Y., Ma, L., Nakamura, N., Miyoshi, K., Sakae, N., Motomura, K., Soejima, N., Yamasaki, R., Hashimoto, T., et al. (2011). Apomorphine treatment in Alzheimer mice promoting amyloid-beta degradation. Ann Neurol *69*, 248-256. 10.1002/ana.22319.

Hobley, L., Li, B., Wood, J.L., Kim, S.H., Naidoo, J., Ferreira, A.S., Khomutov, M., Khomutov, A., Stanley-Wall, N.R., and Michael, A.J. (2017). Spermidine promotes Bacillus subtilis biofilm formation by activating expression of the matrix regulator slrR. J Biol Chem *292*, 12041-12053. 10.1074/jbc.M117.789644.

Hosokawa, M., Arai, T., Masuda-Suzukake, M., Nonaka, T., Yamashita, M., Akiyama, H., and Hasegawa, M. (2012). Methylene blue reduced abnormal tau accumulation in P301L tau transgenic mice. Plos One *7*, e52389. 10.1371/journal.pone.0052389.

Huang, M., Liang, Y., Chen, H., Xu, B., Chai, C., and Xing, P. (2018). The Role of Fluoxetine in Activating Wnt/beta-Catenin Signaling and Repressing beta-Amyloid Production in an Alzheimer Mouse Model. Front Aging Neurosci *10*, 164. 10.3389/fnagi.2018.00164.

Inden, M., Kitamura, Y., Abe, M., Tamaki, A., Takata, K., and Taniguchi, T. (2011). Parkinsonian rotenone mouse model: reevaluation of long-term administration of rotenone in C57BL/6 mice. Biol Pharm Bull *34*, 92-96. 10.1248/bpb.34.92.

Kaizaki, A., Tien, L.T., Pang, Y., Cai, Z., Tanaka, S., Numazawa, S., Bhatt, A.J., and Fan, L.W. (2013). Celecoxib reduces brain dopaminergic neuronaldysfunction, and improves sensorimotor behavioral performance in neonatal rats exposed to systemic lipopolysaccharide. J Neuroinflammation *10*, 45. 10.1186/1742-2094-10-45.

Kali, A., Bhuvaneshwar, D., Charles, P.M., and Seetha, K.S. (2016). Antibacterial synergy of curcumin with antibiotics against biofilm producing clinical bacterial isolates. J Basic Clin Pharm *7*, 93-96. 10.4103/0976-0105.183265.

Kautu, B.B., Carrasquilla, A., Hicks, M.L., Caldwell, K.A., and Caldwell, G.A. (2013). Valproic acid ameliorates C. elegans dopaminergic neurodegeneration with implications for ERK-MAPK signaling. Neurosci Lett *541*, 116-119. 10.1016/j.neulet.2013.02.026.

Keowkase, R., Aboukhatwa, M., Adam, B.L., Beach, J.W., Terry, A.V., Jr., Buccafussco, J.J., and Luo, Y. (2010a). Neuroprotective effects and mechanism of cognitive-enhancing choline analogs JWB 1-84-1 and JAY 2-22-33 in neuronal culture and Caenorhabditis elegans. Mol Neurodegener *5*, 59. 10.1186/1750-1326-5-59.

Keowkase, R., Aboukhatwa, M., and Luo, Y. (2010b). Fluoxetine protects against amyloid-beta toxicity, in part via daf-16 mediated cell signaling pathway, in Caenorhabditis elegans. Neuropharmacology *59*, 358-365. 10.1016/j.neuropharm.2010.04.008.

Kidd, S.K., and Schneider, J.S. (2011). Protective effects of valproic acid on the nigrostriatal dopamine system in a 1-methyl-4-phenyl-1,2,3,6-tetrahydropyridine mouse model of Parkinson's disease. Neuroscience *194*, 189-194. 10.1016/j.neuroscience.2011.08.010.

Koh, S.H., Lee, S.M., Kim, H.Y., Lee, K.Y., Lee, Y.J., Kim, H.T., Kim, J., Kim, M.H., Hwang, M.S., Song, C., et al. (2006). The effect of epigallocatechin gallate on suppressing disease progression of ALS model mice. Neurosci Lett *395*, 103-107. 10.1016/j.neulet.2005.10.056.

Kosuru, R.Y., Roy, A., and Bera, S. (2021). Antagonistic Roles of Gallates and Ascorbic Acid in Pyomelanin Biosynthesis of Pseudomonas aeruginosa Biofilms. Curr Microbiol *78*, 3843-3852. 10.1007/s00284-021-02655-x.

Koutzoumis, D.N., Vergara, M., Pino, J., Buddendorff, J., Khoshbouei, H., Mandel, R.J., and Torres, G.E. (2020). Alterations of the gut microbiota with antibiotics protects dopamine neuron loss and improve motor deficits in a pharmacological rodent model of Parkinson's disease. Exp Neurol *325*, 113159. 10.1016/j.expneurol.2019.113159.

Lalouckova, K., Mala, L., Marsik, P., and Skrivanova, E. (2021). In Vitro Antibacterial Effect of the Methanolic Extract of the Korean Soybean Fermented Product Doenjang against Staphylococcus aureus. Animals (Basel) *11*. 10.3390/ani11082319.

Laloux, C., Derambure, P., Houdayer, E., Jacquesson, J.M., Bordet, R., Destee, A., and Monaca, C. (2008). Effect of dopaminergic substances on sleep/wakefulness in saline- and MPTP-treated mice. J Sleep Res *17*, 101-110. 10.1111/j.1365-2869.2008.00625.x.

Lehtonen, S., Jaronen, M., Vehvilainen, P., Lakso, M., Rudgalvyte, M., Keksa-Goldsteine, V., Wong, G., Courtney, M.J., Koistinaho, J., and Goldsteins, G. (2016). Inhibition of Excessive Oxidative Protein Folding Is Protective in MPP(+) Toxicity-Induced Parkinson's Disease Models. Antioxid Redox Signal *25*, 485-497. 10.1089/ars.2015.6402.

Li, F., Zhang, Y., Lu, X., Shi, J., and Gong, Q. (2019). Icariin improves the cognitive function of APP/PS1 mice via suppressing endoplasmic reticulum stress. Life Sci *234*, 116739. 10.1016/j.lfs.2019.116739.

Liachko, N.F., McMillan, P.J., Guthrie, C.R., Bird, T.D., Leverenz, J.B., and Kraemer, B.C. (2013). CDC7 inhibition blocks pathological TDP-43 phosphorylation and neurodegeneration. Ann Neurol *74*, 39-52. 10.1002/ana.23870.

Lim, G.P., Chu, T., Yang, F., Beech, W., Frautschy, S.A., and Cole, G.M. (2001). The curry spice curcumin reduces oxidative damage and amyloid pathology in an Alzheimer transgenic mouse. J Neurosci *21*, 8370-8377.

Liu, Z., Hamamichi, S., Lee, B.D., Yang, D., Ray, A., Caldwell, G.A., Caldwell, K.A., Dawson, T.M., Smith, W.W., and Dawson, V.L. (2011). Inhibitors of LRRK2 kinase attenuate neurodegeneration and Parkinson-like phenotypes in Caenorhabditis elegans and Drosophila Parkinson's disease models. Hum Mol Genet *20*, 3933-3942. 10.1093/hmg/ddr312.

Locke, C.J., Fox, S.A., Caldwell, G.A., and Caldwell, K.A. (2008). Acetaminophen attenuates dopamine neuron degeneration in animal models of Parkinson's disease. Neurosci Lett *439*, 129-133. 10.1016/j.neulet.2008.05.003.

Lublin, A., Isoda, F., Patel, H., Yen, K., Nguyen, L., Hajje, D., Schwartz, M., and Mobbs, C. (2011). FDA-Approved Drugs that Protect Mammalian Neurons from Glucose Toxicity Slow Aging Dependent on Cbp and Protect Against Proteotoxicity. Plos One *6*. ARTN e27762

10.1371/journal.pone.0027762.

Luo, J., Dong, B., Wang, K., Cai, S., Liu, T., Cheng, X., Lei, D., Chen, Y., Li, Y., Kong, J., and Chen, Y. (2017). Baicalin inhibits biofilm formation, attenuates the quorum sensing-controlled virulence and enhances Pseudomonas aeruginosa clearance in a mouse peritoneal implant infection model. Plos One *12*, e0176883. 10.1371/journal.pone.0176883.

Ma, J., Wang, R., Chen, T., Jiang, S., and Xu, A. (2021). Protective effects of baicalin in a Caenorhabditis elegans model of Parkinson's disease. Toxicol Res (Camb) *10*, 409-417. 10.1093/toxres/tfaa107.

Matlack, K.E., Tardiff, D.F., Narayan, P., Hamamichi, S., Caldwell, K.A., Caldwell, G.A., and Lindquist, S. (2014). Clioquinol promotes the degradation of metal-dependent amyloid-beta (Abeta) oligomers to restore endocytosis and ameliorate Abeta toxicity. Proc Natl Acad Sci U S A *111*, 4013-4018. 10.1073/pnas.1402228111.

McColl, G., Roberts, B.R., Pukala, T.L., Kenche, V.B., Roberts, C.M., Link, C.D., Ryan, T.M., Masters, C.L., Barnham, K.J., Bush, A.I., and Cherny, R.A. (2012). Utility of an improved model of amyloid-beta (Abeta(1)(-)(4)(2)) toxicity in Caenorhabditis elegans for drug screening for Alzheimer's disease. Mol Neurodegener *7*, 57. 10.1186/1750-1326-7-57.

McCormick, A.V., Wheeler, J.M., Guthrie, C.R., Liachko, N.F., and Kraemer, B.C. (2013). Dopamine D2 receptor antagonism suppresses tau aggregation and neurotoxicity. Biol Psychiatry *73*, 464-471. 10.1016/j.biopsych.2012.08.027.

Memariani, H., Memariani, M., and Ghasemian, A. (2019). An overview on anti-biofilm properties of quercetin against bacterial pathogens. World J Microbiol Biotechnol *35*, 143. 10.1007/s11274-019-2719-5.

Miyasaka, T., Xie, C., Yoshimura, S., Shinzaki, Y., Yoshina, S., Kage-Nakadai, E., Mitani, S., and Ihara, Y. (2016). Curcumin improves tau-induced neuronal dysfunction of nematodes. Neurobiol Aging *39*, 69-81. 10.1016/j.neurobiolaging.2015.11.004.

Mocko, J.B., Kern, A., Moosmann, B., Behl, C., and Hajieva, P. (2010). Phenothiazines interfere with dopaminergic neurodegeneration in Caenorhabditis elegans models of Parkinson's disease. Neurobiol Dis *40*, 120-129. 10.1016/j.nbd.2010.03.019.

Ogawa, N., Tanaka, K., Asanuma, M., Kawai, M., Masumizu, T., Kohno, M., and Mori, A. (1994). Bromocriptine protects mice against 6-hydroxydopamine and scavenges hydroxyl free radicals in vitro. Brain Res *657*, 207-213. 10.1016/0006-8993(94)90969-5.

Patil, S.P., Jain, P.D., Ghumatkar, P.J., Tambe, R., and Sathaye, S. (2014). Neuroprotective effect of metformin in MPTP-induced Parkinson's disease in mice. Neuroscience *277*, 747-754. 10.1016/j.neuroscience.2014.07.046.

Pelling, H., Nzakizwanayo, J., Milo, S., Denham, E.L., MacFarlane, W.M., Bock, L.J., Sutton, J.M., and Jones, B.V. (2019). Bacterial biofilm formation on indwelling urethral catheters. Lett Appl Microbiol *68*, 277-293. 10.1111/lam.13144.

Pham, J.V., Yilma, M.A., Feliz, A., Majid, M.T., Maffetone, N., Walker, J.R., Kim, E., Cho, H.J., Reynolds, J.M., Song, M.C., et al. (2019). A Review of the Microbial Production of Bioactive Natural Products and Biologics. Front Microbiol *10*, 1404. 10.3389/fmicb.2019.01404.

Regitz, C., Dussling, L.M., and Wenzel, U. (2014). Amyloid-beta (Abeta(1)(-)(4)(2))-induced paralysis in Caenorhabditis elegans is inhibited by the polyphenol quercetin through activation of protein degradation pathways. Mol Nutr Food Res *58*, 1931-1940. 10.1002/mnfr.201400014.

Rojas, F., Gonzalez, D., Cortes, N., Ampuero, E., Hernandez, D.E., Fritz, E., Abarzua, S., Martinez, A., Elorza, A.A., Alvarez, A., et al. (2015). Reactive oxygen species trigger motoneuron death in non-cell-autonomous models of ALS through activation of c-Abl signaling. Front Cell Neurosci *9*, 203. 10.3389/fncel.2015.00203.

Saewanee, N., Praputpittaya, T., Malaiwong, N., Chalorak, P., and Meemon, K. (2021). Neuroprotective effect of metformin on dopaminergic neurodegeneration and alpha-synuclein aggregation in C. elegans model of Parkinson's disease. Neurosci Res *162*, 13-21. 10.1016/j.neures.2019.12.017.

Sanchis, A., Garcia-Gimeno, M.A., Canada-Martinez, A.J., Sequedo, M.D., Millan, J.M., Sanz, P., and Vazquez-Manrique, R.P. (2019). Metformin treatment reduces motor and neuropsychiatric phenotypes in the zQ175 mouse model of Huntington disease. Exp Mol Med *51*, 1-16. 10.1038/s12276-019-0264-9.

Sandlie, I., Solberg, K., and Kleppe, K. (1980). The effect of caffeine on cell growth and metabolism of thymidine in Escherichia coli. Mutat Res *73*, 29-41. 10.1016/0027-5107(80)90133-5.

Sarkar, S., Raymick, J., Ray, B., Lahiri, D.K., Paule, M.G., and Schmued, L. (2015). Oral Administration of Thioflavin T Prevents Beta Amyloid Plaque Formation in Double Transgenic AD Mice. Curr Alzheimer Res *12*, 837-846. 10.2174/156720501209151019105647.

Saxena, S., Cabuy, E., and Caroni, P. (2009). A role for motoneuron subtype-selective ER stress in disease manifestations of FALS mice. Nat Neurosci *12*, 627-636. 10.1038/nn.2297.

Sedjahtera, A., Gunawan, L., Bray, L., Hung, L.W., Parsons, J., Okamura, N., Villemagne, V.L., Yanai, K., Liu, X.M., Chan, J., et al. (2018). Targeting metals rescues the phenotype in an animal model of tauopathy. Metallomics *10*, 1339-1347. 10.1039/c8mt00153g.

Serra, D.O., Mika, F., Richter, A.M., and Hengge, R. (2016). The green tea polyphenol EGCG inhibits E. coli biofilm formation by impairing amyloid curli fibre assembly and downregulating the biofilm regulator CsgD via the sigma(E) -dependent sRNA RybB. Mol Microbiol *101*, 136-151. 10.1111/mmi.13379.

Shaw, J.D., Brodke, D.S., Williams, D.L., and Ashton, N.N. (2020). Methylene Blue Is an Effective Disclosing Agent for Identifying Bacterial Biofilms on Orthopaedic Implants. J Bone Joint Surg Am *102*, 1784-1791. 10.2106/JBJS.20.00091.

Siddiquia, M.F., Wintersa, H., Maqboolb, F., Qayyumb, S., Singhd, L., Ullahe, I., Rahmanf, Z.U., Adnang, F. and Rehmanh, A. (2019). Tannic acid treatment to deter microbial biofouling in flow cell system and on RO membrane in drip flow reactor. Desalination and Water Treatment *171*, 62-66.

Siles, S.A., Srinivasan, A., Pierce, C.G., Lopez-Ribot, J.L., and Ramasubramanian, A.K. (2013). High-throughput screening of a collection of known pharmacologically active small compounds for identification of Candida albicans biofilm inhibitors. Antimicrob Agents Chemother *57*, 3681-3687. 10.1128/AAC.00680-13.

Singh, D., Gupta, S., Verma, I., Morsy, M.A., Nair, A.B., and Ahmed, A.F. (2021). Hidden pharmacological activities of valproic acid: A new insight. Biomed Pharmacother *142*, 112021. 10.1016/j.biopha.2021.112021.

Sood, A., Warren Beach, J., Webster, S.J., Terry, A.V., and Buccafusco, J.J. (2007). The effects of JWB1-84-1 on memory-related task performance by amyloid Abeta transgenic mice and by young and aged monkeys. Neuropharmacology *53*, 588-600. 10.1016/j.neuropharm.2007.06.028.

Stone, G., Wood, P., Dixon, L., Keyhan, M., and Matin, A. (2002). Tetracycline rapidly reaches all the constituent cells of uropathogenic Escherichia coli biofilms. Antimicrob Agents Chemother *46*, 2458-2461. 10.1128/AAC.46.8.2458-2461.2002.

Suo, H., Wang, P., Tong, J., Cai, L., Liu, J., Huang, D., Huang, L., Wang, Z., Huang, Y., Xu, J., et al. (2015). NRSF is an essential mediator for the neuroprotection of trichostatin A in the MPTP mouse model of Parkinson's disease. Neuropharmacology *99*, 67-78. 10.1016/j.neuropharm.2015.07.015.

Suresh, D., Sabir, S., Yu, T.T., Wenholz, D., Das, T., Black, D.S., and Kumar, N. (2021). Natural Product Rottlerin Derivatives Targeting Quorum Sensing. Molecules *26*. 10.3390/molecules26123745.

Takahashi, H., Kashimura, M., Koiso, H., Kuda, T., and Kimura, B. (2013). Use of ferulic acid as a novel candidate of growth inhibiting agent against Listeria monocytogenes in ready-to-eat food. Food Control *33*, 244-248. 10.1016/j.foodcont.2013.03.013.

Takashi Mori, K.R.-Z., Naoki Koyama, Gary W Arendash, Haruyasu Yamaguchi, Nobuto Kakuda, Yuko Horikoshi-Sakuraba, Jun Tan, Terrence Town (2012). Tannic acid is a natural β-secretase inhibitor that prevents cognitive impairment and mitigates Alzheimer-like pathology in transgenic mice. J Biol Chem *24*. 10.1074/jbc.M111.294025.

Tauffenberger, A., Julien, C., and Parker, J.A. (2013). Evaluation of longevity enhancing compounds against transactive response DNA-binding protein-43 neuronal toxicity. Neurobiol Aging *34*, 2175-2182. 10.1016/j.neurobiolaging.2013.03.014.

Tchantchou, F., Xu, Y., Wu, Y., Christen, Y., and Luo, Y. (2007). EGb 761 enhances adult hippocampal neurogenesis and phosphorylation of CREB in transgenic mouse model of Alzheimer's disease. FASEB J *21*, 2400-2408. 10.1096/fj.06-7649com.

Thongbhubate, K., Nakafuji, Y., Matsuoka, R., Kakegawa, S., and Suzuki, H. (2021). Effect of Spermidine on Biofilm Formation in Escherichia coli K-12. J Bacteriol *203*. 10.1128/JB.00652-20.

Tsai, C.W., Tsai, R.T., Liu, S.P., Chen, C.S., Tsai, M.C., Chien, S.H., Hung, H.S., Lin, S.Z., Shyu, W.C., and Fu, R.H. (2017). Neuroprotective Effects of Betulin in Pharmacological and Transgenic Caenorhabditis elegans Models of Parkinson's Disease. Cell Transplant *26*, 1903-1918. 10.1177/0963689717738785.

Tzeng, S.R., Huang, Y.W., Zhang, Y.Q., Yang, C.Y., Chien, H.S., Chen, Y.R., Yu, S.L., Chen, C.S., and Chiu, H.C. (2020). A Celecoxib Derivative Eradicates Antibiotic-Resistant Staphylococcus aureus and Biofilms by Targeting YidC2 Translocase. Int J Mol Sci *21*. 10.3390/ijms21239312.

Umeda, T., Tanaka, A., Sakai, A., Yamamoto, A., Sakane, T., and Tomiyama, T. (2018). Intranasal rifampicin for Alzheimer's disease prevention. Alzheimers Dement (N Y) *4*, 304-313. 10.1016/j.trci.2018.06.012.

Vaccaro, A., Patten, S.A., Aggad, D., Julien, C., Maios, C., Kabashi, E., Drapeau, P., and Parker, J.A. (2013). Pharmacological reduction of ER stress protects against TDP-43 neuronal toxicity in vivo. Neurobiol Dis *55*, 64-75. 10.1016/j.nbd.2013.03.015.

Vaccaro, A., Patten, S.A., Ciura, S., Maios, C., Therrien, M., Drapeau, P., Kabashi, E., and Parker, J.A. (2012a). Methylene blue protects against TDP-43 and FUS neuronal toxicity in C. elegans and D. rerio. Plos One *7*, e42117. 10.1371/journal.pone.0042117.

Vaccaro, A., Tauffenberger, A., Ash, P.E., Carlomagno, Y., Petrucelli, L., and Parker, J.A. (2012b). TDP-1/TDP-43 regulates stress signaling and age-dependent proteotoxicity in Caenorhabditis elegans. PLoS Genet *8*, e1002806. 10.1371/journal.pgen.1002806.

Varma, H., Cheng, R., Voisine, C., Hart, A.C., and Stockwell, B.R. (2007). Inhibitors of metabolism rescue cell death in Huntington's disease models. Proc Natl Acad Sci U S A *104*, 14525-14530. 10.1073/pnas.0704482104.

Ved, R., Saha, S., Westlund, B., Perier, C., Burnam, L., Sluder, A., Hoener, M., Rodrigues, C.M., Alfonso, A., Steer, C., et al. (2005). Similar patterns of mitochondrial vulnerability and rescue induced by genetic modification of alpha-synuclein, parkin, and DJ-1 in Caenorhabditis elegans. J Biol Chem *280*, 42655-42668. 10.1074/jbc.M505910200.

Verma, R., Gurumurthy, M., Yeo, B.C.M., Lu, Q., Naftalin, C.M., and Paton, N.I. (2021). Effects of increasing concentrations of rifampicin on different Mycobacterium tuberculosis lineages in a Whole blood Bactericidal Activity Assay. Antimicrob Agents Chemother, AAC0169921. 10.1128/AAC.01699-21.

Vieira, F.G., Ping, Q., Moreno, A.J., Kidd, J.D., Thompson, K., Jiang, B., Lincecum, J.M., Wang, M.Z., De Zutter, G.S., Tassinari, V.R., et al. (2015). Guanabenz Treatment Accelerates Disease in a Mutant SOD1 Mouse Model of ALS. Plos One *10*, e0135570. 10.1371/journal.pone.0135570.

Viszwapriya, D., Subramenium, G.A., Prithika, U., Balamurugan, K., and Pandian, S.K. (2016). Betulin inhibits virulence and biofilm of Streptococcus pyogenes by suppressing ropB core regulon, sagA and dltA. Pathog Dis *74*. 10.1093/femspd/ftw088.

Voisine, C., Varma, H., Walker, N., Bates, E.A., Stockwell, B.R., and Hart, A.C. (2007). Identification of potential therapeutic drugs for huntington's disease using Caenorhabditis elegans. Plos One *2*, e504. 10.1371/journal.pone.0000504.

Wang, C., Lau, C.Y., Ma, F., and Zheng, C. (2021a). Genome-wide screen identifies curli amyloid fibril as a bacterial component promoting host neurodegeneration. Proc Natl Acad Sci U S A *118*. 10.1073/pnas.2106504118.

Wang, I.F., Guo, B.S., Liu, Y.C., Wu, C.C., Yang, C.H., Tsai, K.J., and Shen, C.K. (2012). Autophagy activators rescue and alleviate pathogenesis of a mouse model with proteinopathies of the TAR DNA-binding protein 43. Proc Natl Acad Sci U S A *109*, 15024-15029. 10.1073/pnas.1206362109.

Wang, N., Zhou, Y., Zhao, L., Wang, C., Ma, W., Ge, G., Wang, Y., Ullah, I., Muhammad, F., Alwayli, D., et al. (2020). Ferulic acid delayed amyloid beta-induced pathological symptoms by autophagy pathway via a fasting-like effect in Caenorhabditis elegans. Food Chem Toxicol *146*, 111808. 10.1016/j.fct.2020.111808.

Wang, N.Y., Li, J.N., Liu, W.L., Huang, Q., Li, W.X., Tan, Y.H., Liu, F., Song, Z.H., Wang, M.Y., Xie, N., et al. (2021b). Ferulic Acid Ameliorates Alzheimer's Disease-like Pathology and Repairs Cognitive Decline by Preventing Capillary Hypofunction in APP/PS1 Mice. Neurotherapeutics *18*, 1064-1080. 10.1007/s13311-021-01024-7.

Wong, S.Q., Pontifex, M.G., Phelan, M.M., Pidathala, C., Kraemer, B.C., Barclay, J.W., Berry, N.G., O'Neill, P.M., Burgoyne, R.D., and Morgan, A. (2018). alpha-Methyl-alpha-phenylsuccinimide ameliorates neurodegeneration in a C. elegans model of TDP-43 proteinopathy. Neurobiol Dis *118*, 40-54. 10.1016/j.nbd.2018.06.013.

Wu, J., Xu, H., Tang, W., Kopelman, R., Philbert, M.A., and Xi, C. (2009). Eradication of bacteria in suspension and biofilms using methylene blue-loaded dynamic nanoplatforms. Antimicrob Agents Chemother *53*, 3042-3048. 10.1128/AAC.01604-08.

Wu, Y., Park, K.C., Choi, B.G., Park, J.H., and Yoon, K.S. (2016). The Antibiofilm Effect of Ginkgo biloba Extract Against Salmonella and Listeria Isolates from Poultry. Foodborne Pathog Dis *13*, 229-238. 10.1089/fpd.2015.2072.

Wu, Y., Wu, Z., Butko, P., Christen, Y., Lambert, M.P., Klein, W.L., Link, C.D., and Luo, Y. (2006). Amyloid-beta-induced pathological behaviors are suppressed by Ginkgo biloba extract EGb 761 and ginkgolides in transgenic Caenorhabditis elegans. J Neurosci *26*, 13102-13113. 10.1523/JNEUROSCI.3448-06.2006.

Xiao, L., Li, H., Zhang, J., Yang, F., Huang, A., Deng, J., Liang, M., Ma, F., Hu, M., and Huang, Z. (2014). Salidroside protects Caenorhabditis elegans neurons from polyglutamine-mediated toxicity by reducing oxidative stress. Molecules *19*, 7757-7769. 10.3390/molecules19067757.

Xin, L., Yamujala, R., Wang, Y., Wang, H., Wu, W.H., Lawton, M.A., Long, C., and Di, R. (2013). Acetylcholineestarase-inhibiting alkaloids from Lycoris radiata delay paralysis of amyloid beta-expressing transgenic C. elegans CL4176. Plos One *8*, e63874. 10.1371/journal.pone.0063874.

Yamada, K.J., Heim, C.E., Xi, X., Attri, K.S., Wang, D., Zhang, W., Singh, P.K., Bronich, T.K., and Kielian, T. (2020). Monocyte metabolic reprogramming promotes pro-inflammatory activity and Staphylococcus aureus biofilm clearance. PLoS Pathog *16*, e1008354. 10.1371/journal.ppat.1008354.

Yang, X., Zhang, M., Dai, Y., Sun, Y., Aman, Y., Xu, Y., Yu, P., Zheng, Y., Yang, J., and Zhu, X. (2020). Spermidine inhibits neurodegeneration and delays aging via the PINK1-PDR1-dependent mitophagy pathway in C. elegans. Aging (Albany NY) *12*, 16852-16866. 10.18632/aging.103578.

Yao, C., Johnson, W.M., Gao, Y., Wang, W., Zhang, J., Deak, M., Alessi, D.R., Zhu, X., Mieyal, J.J., Roder, H., et al. (2013). Kinase inhibitors arrest neurodegeneration in cell and C. elegans models of LRRK2 toxicity. Hum Mol Genet *22*, 328-344. 10.1093/hmg/dds431.

You, Z., Ran, X., Dai, Y., and Ran, Y. (2018). Clioquinol, an alternative antimicrobial agent against common pathogenic microbe. J Mycol Med *28*, 492-501. 10.1016/j.mycmed.2018.03.007.

You, Z., Zhang, C., and Ran, Y. (2020). The effects of clioquinol in morphogenesis, cell membrane and ion homeostasis in Candida albicans. BMC Microbiol *20*, 165. 10.1186/s12866-020-01850-3.

Zaidi, S., Singh, S.L., and Khan, A.U. (2020). Exploring antibiofilm potential of bacitracin against streptococcus mutans. Microb Pathog *149*, 104279. 10.1016/j.micpath.2020.104279.

Zhang, C., Liu, Z., Bunker, E., Ramirez, A., Lee, S., Peng, Y., Tan, A.C., Eckhardt, S.G., Chapnick, D.A., and Liu, X. (2017). Sorafenib targets the mitochondrial electron transport chain complexes and ATP synthase to activate the PINK1-Parkin pathway and modulate cellular drug response. J Biol Chem *292*, 15105-15120. 10.1074/jbc.M117.783175.

Zhang, D., Anantharam, V., Kanthasamy, A., and Kanthasamy, A.G. (2007). Neuroprotective effect of protein kinase C delta inhibitor rottlerin in cell culture and animal models of Parkinson's disease. J Pharmacol Exp Ther *322*, 913-922. 10.1124/jpet.107.124669.

Zhang, H., Su, Y., Sun, Z., Chen, M., Han, Y., Li, Y., Dong, X., Ding, S., Fang, Z., Li, W., and Li, W. (2021). Ginsenoside Rg1 alleviates Abeta deposition by inhibiting NADPH oxidase 2 activation in APP/PS1 mice. J Ginseng Res *45*, 665-675. 10.1016/j.jgr.2021.03.003.

Zhang, S.Q., Obregon, D., Ehrhart, J., Deng, J., Tian, J., Hou, H., Giunta, B., Sawmiller, D., and Tan, J. (2013). Baicalein reduces beta-amyloid and promotes nonamyloidogenic amyloid precursor protein processing in an Alzheimer's disease transgenic mouse model. J Neurosci Res *91*, 1239-1246. 10.1002/jnr.23244.

Zhang, W., He, H., Song, H., Zhao, J., Li, T., Wu, L., Zhang, X., and Chen, J. (2016). Neuroprotective Effects of Salidroside in the MPTP Mouse Model of Parkinson's Disease: Involvement of the PI3K/Akt/GSK3beta Pathway. Parkinsons Dis *2016*, 9450137. 10.1155/2016/9450137.

Zhao, W.X., Zhang, J.H., Cao, J.B., Wang, W., Wang, D.X., Zhang, X.Y., Yu, J., Zhang, Y.Y., Zhang, Y.Z., and Mi, W.D. (2017). Acetaminophen attenuates lipopolysaccharide-induced cognitive impairment through antioxidant activity. J Neuroinflammation *14*, 17. 10.1186/s12974-016-0781-6.

Zhou, T., Zhu, M., and Liang, Z. (2018). (-)-Epigallocatechin-3-gallate modulates peripheral immunity in the MPTP-induced mouse model of Parkinson's disease. Mol Med Rep *17*, 4883-4888. 10.3892/mmr.2018.8470.
